# Integrated Transcriptomic and Epigenomic Analysis of Primary Human Lung Epithelial Cell Differentiation

**DOI:** 10.1371/journal.pgen.1003513

**Published:** 2013-06-20

**Authors:** Crystal N. Marconett, Beiyun Zhou, Megan E. Rieger, Suhaida A. Selamat, Mickael Dubourd, Xiaohui Fang, Sean K. Lynch, Theresa Ryan Stueve, Kimberly D. Siegmund, Benjamin P. Berman, Zea Borok, Ite A. Laird-Offringa

**Affiliations:** 1 Department of Surgery, Keck School of Medicine, University of Southern California, Los Angeles, California, United States of America; 2 Department of Biochemistry and Molecular Biology, Keck School of Medicine, University of Southern California, Los Angeles, California, United States of America; 3 Norris Comprehensive Cancer Center, Keck School of Medicine, University of Southern California, Los Angeles, California, United States of America; 4 Will Rogers Institute Pulmonary Research Center and Division of Pulmonary, Critical Care and Sleep Medicine, Department of Medicine, Keck School of Medicine, University of Southern California, Los Angeles, California, United States of America; 5 Cardiovascular Research Institute, University of California San Francisco, San Francisco, California, United States of America; 6 Department of Medicine/Pathology, University of California San Francisco, San Francisco, California, United States of America; 7 Department of Product Engineering, Division of Manufacturing Operations, MAXIM Integrated Products, Sunnyvale, California, United States of America; 8 Department of Preventive Medicine, Keck School of Medicine, University of Southern California, Los Angeles, California, United States of America; 9 University of Southern California Epigenome Center, Keck School of Medicine, University of Southern California, Los Angeles, California, United States of America; Medical College of Georgia, United States of America

## Abstract

Elucidation of the epigenetic basis for cell-type specific gene regulation is key to gaining a full understanding of how the distinct phenotypes of differentiated cells are achieved and maintained. Here we examined how epigenetic changes are integrated with transcriptional activation to determine cell phenotype during differentiation. We performed epigenomic profiling in conjunction with transcriptomic profiling using *in vitro* differentiation of human primary alveolar epithelial cells (AEC). This model recapitulates an *in vivo* process in which AEC transition from one differentiated cell type to another during regeneration following lung injury. Interrogation of histone marks over time revealed enrichment of specific transcription factor binding motifs within regions of changing chromatin structure. Cross-referencing of these motifs with pathways showing transcriptional changes revealed known regulatory pathways of distal alveolar differentiation, such as the WNT and transforming growth factor beta (TGFB) pathways, and putative novel regulators of adult AEC differentiation including hepatocyte nuclear factor 4 alpha (HNF4A), and the retinoid X receptor (RXR) signaling pathways. Inhibition of the RXR pathway confirmed its functional relevance for alveolar differentiation. Our incorporation of epigenetic data allowed specific identification of transcription factors that are potential direct upstream regulators of the differentiation process, demonstrating the power of this approach. Integration of epigenomic data with transcriptomic profiling has broad application for the identification of regulatory pathways in other models of differentiation.

## Author Summary

Understanding the role of epigenetic control of gene expression is critical to the full description of biological processes, such as development and regeneration. Herein we utilize the differentiation of cells from the distal lung to gain insight into the correlation between the epigenetic landscape, molecular signaling events, and eventual changes in transcription and phenotype. We found that by integrating epigenetic profiling with whole genome transcriptomic data we were able to determine which molecular signaling events were activated and repressed during adult alveolar epithelial cell differentiation, and we identified epigenetic changes that contributed to these changes. Furthermore, we validated the role of one of these predicted but not previously identified pathways, retinoid X receptor signaling, in this process.

## Introduction

Over the past two decades the relationship between gene expression and chromatin structure has been increasingly recognized [1]–[4]. Elucidation of the histone code and subsequent insights into the functional implications of post-translational modifications of histone tails have begun to provide a mechanistic understanding of the role that chromatin context plays in gene expression. One of the most widely studied histone marks of active gene transcription is acetylation of lysine residues in the N-terminal tail of histone H3. Acetylation of Lysines 9 and 14 (H3K9/14^Ac^), found at promoters and enhancers of actively transcribed genes [5]–[7], serves as a docking point for chromatin remodeling complexes that open chromatin, facilitating transcriptional activation [8]–[10]. In contrast, trimethylation of lysine 27 of histone H3 (H3K27^me3^) confers repression through binding of the polycomb repressive complex (PRC1/2) and chromatin compaction [11]–[13]. The H3K9/14^Ac^ and H3K27^me3^ marks usually occur in distinct cell-type specific genomic regions. Many studies have examined the differentiation of stem cells into a variety of differentiated cell types, in processes that traverse large phenotypic (and presumably epigenetic) distances [14]. However, using stem cells to dissect the mechanism(s) by which the differentiated epigenotype is reached may be challenging due to the distant relationship between the starting and resulting cell populations. In contrast, examining epigenetic differences between two closely related yet phenotypically distinct cell types might offer more straightforward insights into the relationship between epigenetic changes and the establishment of new expression patterns. Isolated distal lung epithelial cells offer a compelling model system; primary human alveolar epithelial type 2 (AT2) cells can be purified in large numbers from remnant transplant lung, and can be differentiated *in vitro* in a manner closely mimicking both normal maintenance and regeneration following lung injury [15], [16].

The distal lung alveolar epithelium consists of two major cell types: cuboidal surfactant-producing AT2 cells and elongated type 1 (AT1) cells that facilitate gas exchange. AT2 cells are the implicated precursors of AT1 cells, and through differentiation restore function of damaged distal lung epithelium [17], [18]. This phenotypic transition can be recapitulated *in vitro* when purified primary AT2 cells are plated under defined cell culture conditions [19], [20]. Previous studies using purified rat AT2 cells showed that AT2 cell-specific markers, such as surfactant protein C and A (SFTPC, SFTPA1), decrease with time in culture, while AT1 cell markers, such as aquaporin 5 (AQP5 [21], [22]), caveolin 1 (CAV1, [23], [24]) and podoplanin (PDPN, [25], [26]) increase over time [27]. These expression changes are accompanied by profound changes in cellular morphology and function [28]. Recent advances have allowed for the isolation of human AT2 cells from remnant human transplant lungs [15], [29].

Differentiation of purified primary human AT2 cells into AT1-like cells represents a unique kinetic model system to study the process of epithelial cell differentiation without the caveats of cell line immortalization or mixed tissue analysis. Here, we simultaneously profiled the transcriptomic and epigenomic changes of differentiating human alveolar epithelial cells (AEC). Detailed integrated analysis facilitated identification of regulatory networks and participating transcription factors, pointing to roles for known and novel signaling pathways in distal lung epithelial differentiation. To our knowledge this is the first integrated analysis of human primary epithelial cell differentiation.

## Results

### Transcriptomic profiling of human alveolar epithelial type 2 cells during differentiation

AT2 cells were isolated from the lungs of three non-smoker donors (Figure S1A) and plated on collagen-coated polytetrafluoroethylene membranes. Purity was verified by immunostaining for AT2 cell-specific markers pro-SFTPC and transcription factor NK2 homeobox 1 (NKX2-1), as well as hematopoietic marker protein tyrosine phosphatase, receptor type, C (PTPRC, previously CD45) and mesenchymal marker vimentin (VIM) to check for contaminating cell types. Pro-SFTPC-positive cells averaged 86% purity (Figure S1A–1B). Over the course of 8 days the cells underwent differentiation into AT1-like cells, forming confluent monolayers with tight junctions expressing tight junction protein 1 (TJP1, previously ZO-1) and showing downregulation of *SFTPC*, upregulation of *AQP5*, and establishment of transepithelial resistance [27], [30], [31] (Figure S1C–1E).

**Figure 1:**
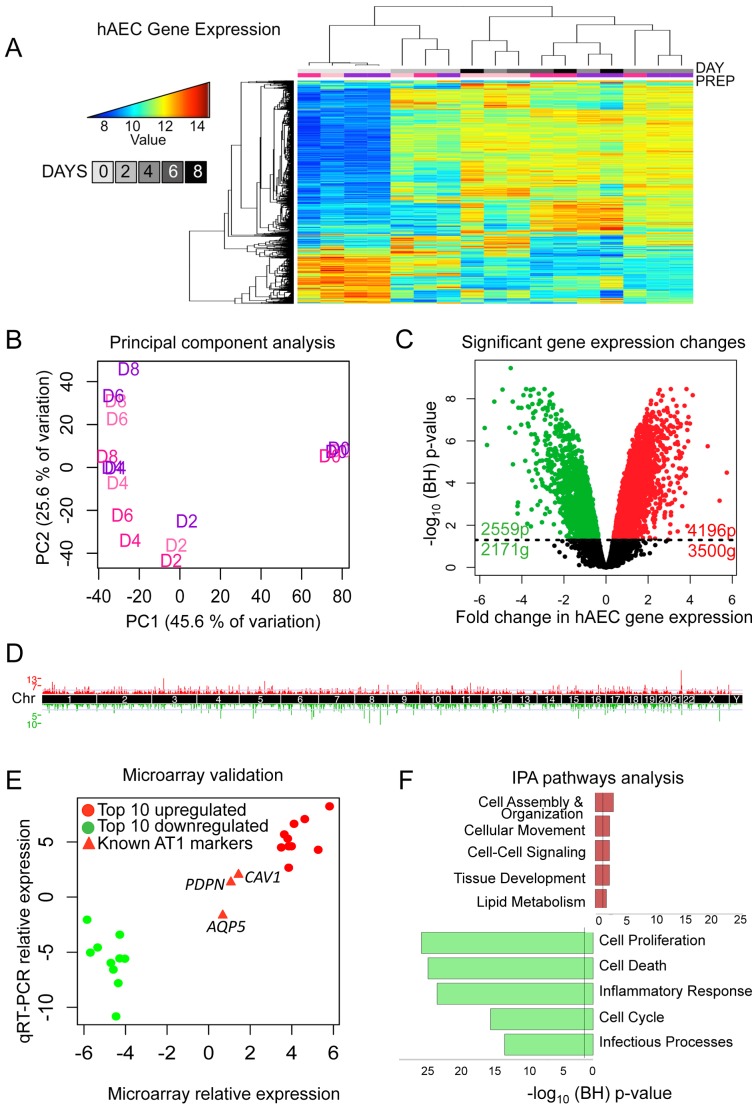
Transcriptomic profiling of human AEC differentiation. A) Heatmap of top 5% variant-VSN normalized gene expression probes. Blue = low expression, red = high expression. DAY = number of days AT2 cells were allowed to differentiate. “Prep” = donor lung origin by color (Figure S1). B) Principal component analysis of normalized hAEC samples. Samples color coded by donor lung as in (A). C) Significant changes in hAEC gene expression. Black line = BH-adjusted cutoff (FDR adjusted p≤0.05) calculated between D0 and D8. 20 genes show both significant up and downregulation for probes in different locations of the gene. D) Manhattan plot of differentially expressed genes. X-axis = chromosomal location, Y-axis = number of genes in each 2 MB region. E) qRT-PCR validation of microarray, data expressed in log_2_-fold change of differences between D0 and D8. Circles = top 10 up- and down-regulated genes, triangles = known AT1 cell differentiation markers (*AQP5, PDPN, CAV1*). F) IPA of significantly up- or down-regulated genes. Bars expressed as log_10_-BH corrected p-values of enrichment for pathway members in significant list against RefSeq db38 background. Whole figure: Red = upregulated, green = downregulated.

Raw expression data across all samples had similar distributions (Figure S2), GEO accession #GSE38571. Thus, all samples, including a technical duplicate each of day 0 (D0) and day 4 (D4), were included in normalization (Figure S3). The relationship between gene expression profiles was examined by unsupervised hierarchical clustering using the top 5% of genes most variant across the dataset. Samples from different lungs clustered together based largely on the timing of differentiation, with the D0 and D2 samples each grouping together (Figure 1A). Importantly, the sample dendrogram indicates the major branch point is between D2 and D4, which has been observed previously as the point in time when the largest shift from AT2 to AT1 phenotype occurs in gene expression and morphology [32]. Clustering did not change using alternate cutoffs in the number of genes (top 2%, top 10%) (Figure S4). A two-dimensional principal component analysis plot showed that time in culture corresponded to PC1 (46% of total variation in the samples) (Figure 1B), suggesting that the differentiation process contributed most to inter-sample variation.

A linear model was fit over the 5 time points (D0, D2, D4, D6, and D8) excluding the technical duplicates and a moderated t-test was used to determine significance of changes in gene expression from D0 (AT2 cells) to D8 (AT1-like cells). 6755 probes (5651 genes) showed statistically significant changes in gene expression; 4196 upregulated probes (3500 genes) and 2559 downregulated probes (2171 genes) (Figure 1C). These changes were distributed throughout the genome (Figure 1D). qRT-PCR of the top 10 up- and down-regulated genes showed a high degree of correlation with the microarray expression results (Figure 1E, Figure S5). Genes known to become activated during AT2 to AT1 cell differentiation (*PDPN*, *CAV1*, and *AQP5*) were also assessed using qRT-PCR and values were plotted for comparison alongside the top differentially expressed genes (Figure 1E, red triangles).

Ingenuity Pathways Analysis (IPA) revealed that the top upregulated pathways were cell assembly and organization, cell movement, cell-cell signaling, tissue development, and lipid metabolism, all of which are consistent with AT2 cells differentiating into larger, flat AT1 cells, while the most downregulated pathways pertained to cell proliferation, cell death, inflammatory response, cell cycle and infectious processes (Figure 1F). Signaling pathways in the upregulated networks included those of v-akt murine thymoma viral oncogene homolog (*AKT*), protein kinase C (*PRKC*), and the *RAS* family *RAB* genes, implicated in endocytosis, while FBJ murine osteosarcoma viral gene homolog (*FOS*) was the center of interconnectivity in the top downregulated network (Figure S6). Analysis using another pathway prediction program DAVID yielded results consistent with IPA (Figure S7).

### Comparative transcriptomic profiling of human and rat alveolar epithelial cell differentiation

**Figure 2:**
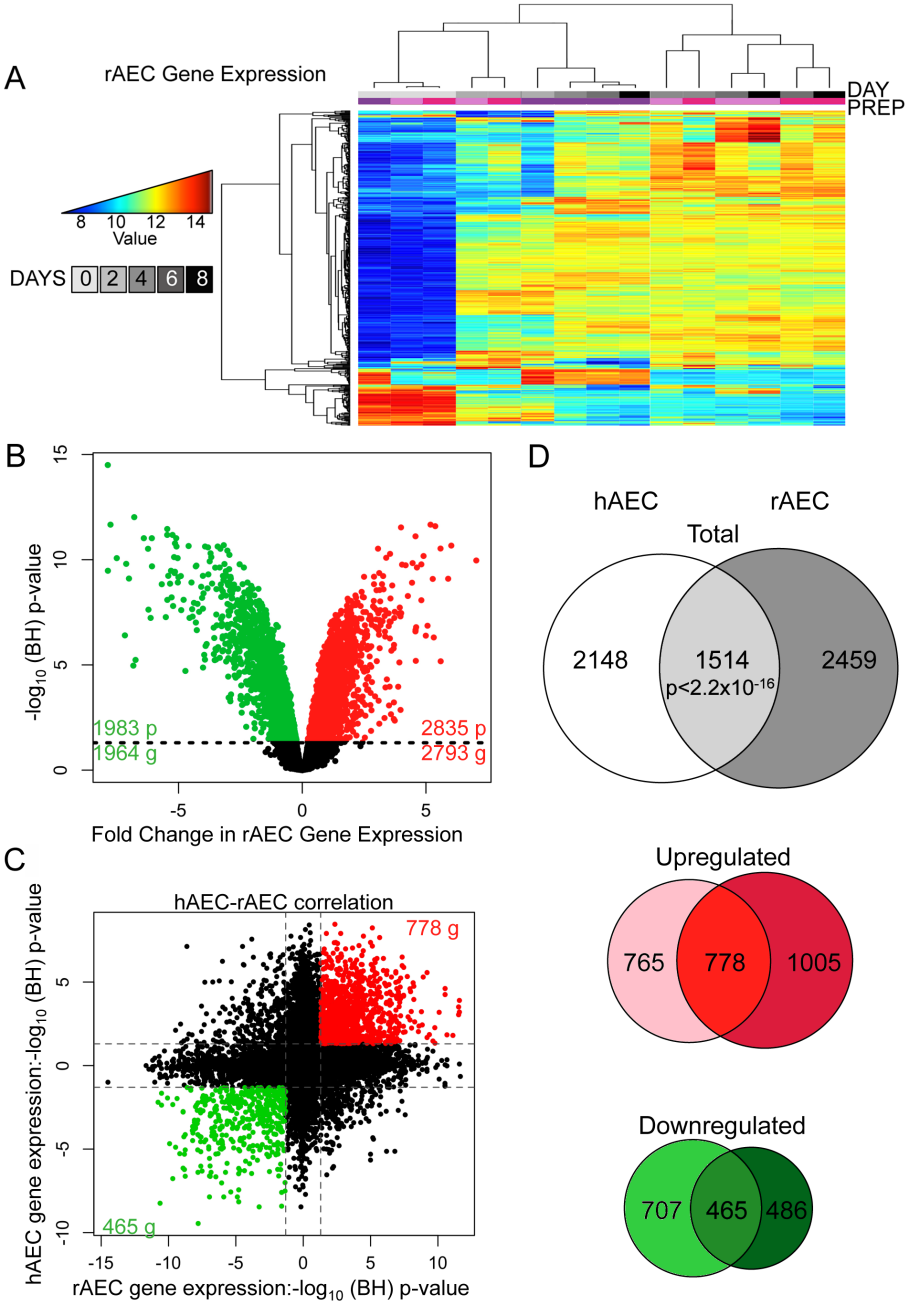
Comparative transcriptomic profiling of human and rat AEC differentiation. A) Heatmap of top 2% of variant-VSN normalized gene expression probes in rat AEC. Blue = low expression, red = high expression. Prep = separate rAEC purifications. B) Significant changes in rAEC gene expression: red = upregulated, green = downregulated, black line = BH-adjusted cutoff for significance (FDR adjusted p≤0.05) calculated between D0 and D8. C) Correlation between rAEC and hAEC statistically significant genes. Data points expressed as significance of change between D0 and D8. Direction of change derived from increase or decrease in gene expression. Red = statically significant upregulated genes in both hAEC and rAEC, green = statistically significant downregulated genes in both hAEC and rAEC. Dotted lines = BH-adjusted cutoff for significance (p.adjusted≤0.05) calculated between D0 and D8. D) Venn diagram of statistically significant gene overlap between hAEC and rAEC (Top), genes upregulated between hAEC and rAEC (Middle), and genes downregulated between hAEC and rAEC (Bottom). 271 genes were significant in both species but expression changed in opposite directions. In all three diagrams: pale color = hAEC-specific statistically significant gene expression changes, medium color = statistically significant overlap in both rAEC and hAEC, dark color = rAEC-specific statistically significant gene expression changes.

Differentiation of AT2 cells into AT1 cells has been extensively studied in the rat [33], [19]. To compare differentiation between rat AEC (rAEC) and human AEC (hAEC), purified rat AT2 cells were cultured under differentiation-permissive conditions and RNA was subjected to whole genome profiling. Technical variation within raw data was minimal (Figure S8). Data was preprocessed and clustered similarly to the human expression arrays (Figure S9). Purified AT2 cells clustered separately from AEC differentiating toward the AT1 cell phenotype (Figure 2A, Figure S10). We observed 4860 significantly changing probes corresponding to 4799 genes, with 2835 probes (2793 genes) upregulated and 1983 probes (1964 genes) downregulated (Figure 2B). For comparison, human and rat expression arrays were subset to include probes represented on both arrays; 13173 genes were represented in both species. This resulted in 3973 and 3662 statistically significant gene expression alterations in rat and human AEC respectively. Of these statistically significant gene sets derived separately from both species, 1514 genes were significantly differentially expressed during both human and rat AT2 to AT1 differentiation (Figure 2C–2D, p-value <2.2×10^−16^). Separating significantly up- or downregulated genes yielded similar degrees of overlap (Figure 2D). Therefore, differentiation of AT2 into AT1-like cells involves coordinated changes in thousands of genes, many of which occur in both human and rat. IPA analysis of genes concordantly changing in rat and human AT2 cell differentiation identified many similar altered pathways in both DAVID and IPA results (Figure 1E, Figure S11A). The most significant network from this joint analysis centered on genes involved in lipid metabolism (Figure S11B).

The non-overlapping subsets of rAEC and hAEC genes were also of interest because they could indicate potential interspecies variation. While cell cycle control networks featured more prominently in the rat IPA networks, possibly reflecting species-specific differences, IPA of the hAEC and rAEC-specific gene sets revealed that 3 of the top 5 molecular signaling processes were identical (Figure S12, Figure S13), suggesting that different genes in a similar pathway were modulated in the two species to achieve similar effects. The top signaling network in hAEC-specific genes, cell growth and proliferation, centered on *HNF4A* and was the third most significant pathway in rAEC-specific alterations. *TGFB* featured prominently in rAEC-specific and in overall human IPA analysis.

### Profiling of altered chromatin states during AT2 to AT1 cell differentiation

**Figure 3:**
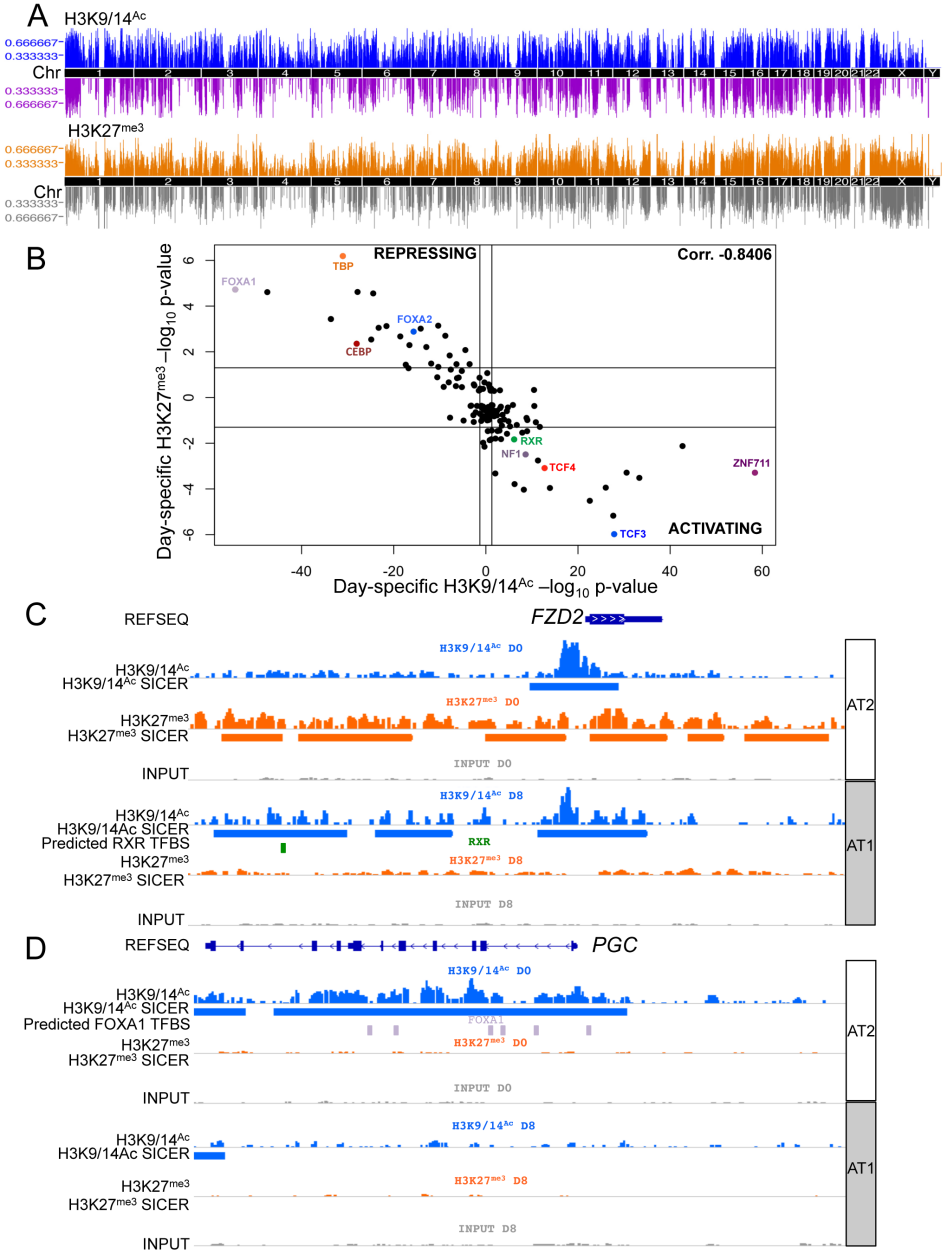
Chromatin changes during AEC differentiation. A) Manhattan plot of differential chromatin changes. X-axis = chromosomal location, Y-axis = number of cell type-specific chromatin changes within 2 MB region. Upper panel = H3K9/14^Ac^ changes, blue = AT2 cell-specific acetylation, purple = AT1 cell-specific acetylation. Lower panel = H3K27^me3^ changes, orange = AT2 cell-specific methylation, grey = AT1 cell-specific methylation. B) 135 TFBS enrichment in domains of chromatin change from HOMER. X-axis = H3K9/14^Ac^, Y-axis = H3K27^me3^ enrichment. AT2 enrichment is shown as the log_10_ TFBS p-value, AT1 enrichment is shown as the −log_10_ TFBS p-value. C) Example of chromatin changes at an upregulated gene, *FZD2*, using IGV to visualize chromatin tracks. Blue = H3K9/14^Ac^ raw reads and SICER peaks called, green = predicted RXR binding site from HOMER analysis. D) Example of downregulated gene expression at the *PGC* gene locus. Lavender = predicted FOXA1 binding sites from HOMER analysis. AT2 = AEC chromatin signature (D0), AT1 = AEC chromatin signature (D8).

Using subsets of the same batches of human AT2 cells (D0) and fully differentiated AT1-like cells (D8) used for expression analyses, we assessed alterations to the chromatin environment by chromatin immunoprecipitation followed by sequencing (ChIP-seq). Specifically, H3K9/14^Ac^ was used to interrogate active promoter and enhancer elements, and H3K27^me3^ was used to assess the repressed chromatin state. Peaks were called using both the Spatial clustering approach for the Identification of ChIP Enriched Regions (SICER) and the Model-based Analysis of ChIP-Seq (MACS) methods. Each analysis revealed thousands of enriched regions, with a significant fraction (20–40%) shared between D0 and D8 and the remainder of them specific to D0 or D8 (Figure S14). Changes in chromatin were distributed throughout the genome (Figure 3A). The transcription factor binding site (TFBS) predictor program Hypergeometric Optimization of Motif EnRichment (HOMER) was used to identify conserved sequence motifs enriched within day-specific chromatin marks [34]. For each of the 135 TFBSs in HOMER, the strongest association with either D0 or D8 H3K9/14^Ac^ from SICER-called peaks was determined, and likewise for D0 and D8 H3K27^me3^ (Table S1). We then plotted the strongest H3K9/14^Ac^ significance level versus the strongest H3K27^me3^ significance level for all TFBSs (with negative significance values representing D0 and positive D8) (Figure 3B), revealing a striking correlation – those motifs associated with regions losing H3K9/14^Ac^ from D0 to D8 were consistently associated with regions gaining H3K27^me3^ from D0 to D8. Conversely, motifs associated with regions gaining H3K9/14^Ac^ were consistently associated with regions losing H3K27^me3^. While this basic trend was expected based on the antagonism between these two marks, the identification of so many transcription factors apparently involved in regulation of both these marks was surprising. Some of the stronger associations with activating chromatin changes included motifs for zinc finger protein 711 (ZNF711), transcription factor 3 (TCF3 or E2A), TCF4 (a WNT signaling target), and RXR. TFBSs strongly associated with repressive chromatin changes included those for forkhead box (FOX) proteins FOXA1 and FOXA2, TATA-box binding protein TBP, and CCAAT/enhancer-binding protein CEBP. Two example loci illustrate the location of particular motifs within differentially marked chromatin regions – an upregulated gene, frizzled family receptor 2 (*FZD2*), which shows spreading acetylation and loss of H3K27^me3^, is predicted to have an RXR site within activating chromatin marks (Figure 3C), while a downregulated gene, progastricin (*PGC*), shows loss of acetylation but no gain of H3K27^me3^ and is predicted to have numerous FOXA1 sites within silenced regions (Figure 3D). These two examples illustrate that different combinations of marks might be found on activated and repressed genes. To further investigate this, we examined the association between transcriptomic and epigenetic changes by analyzing the relationship between changes in gene expression and all possible combinations of H3K9/14^Ac^ and H3K27^me3^ from D0 to D8.

### Integration of gene expression data with epigenetic alterations

**Figure 4:**
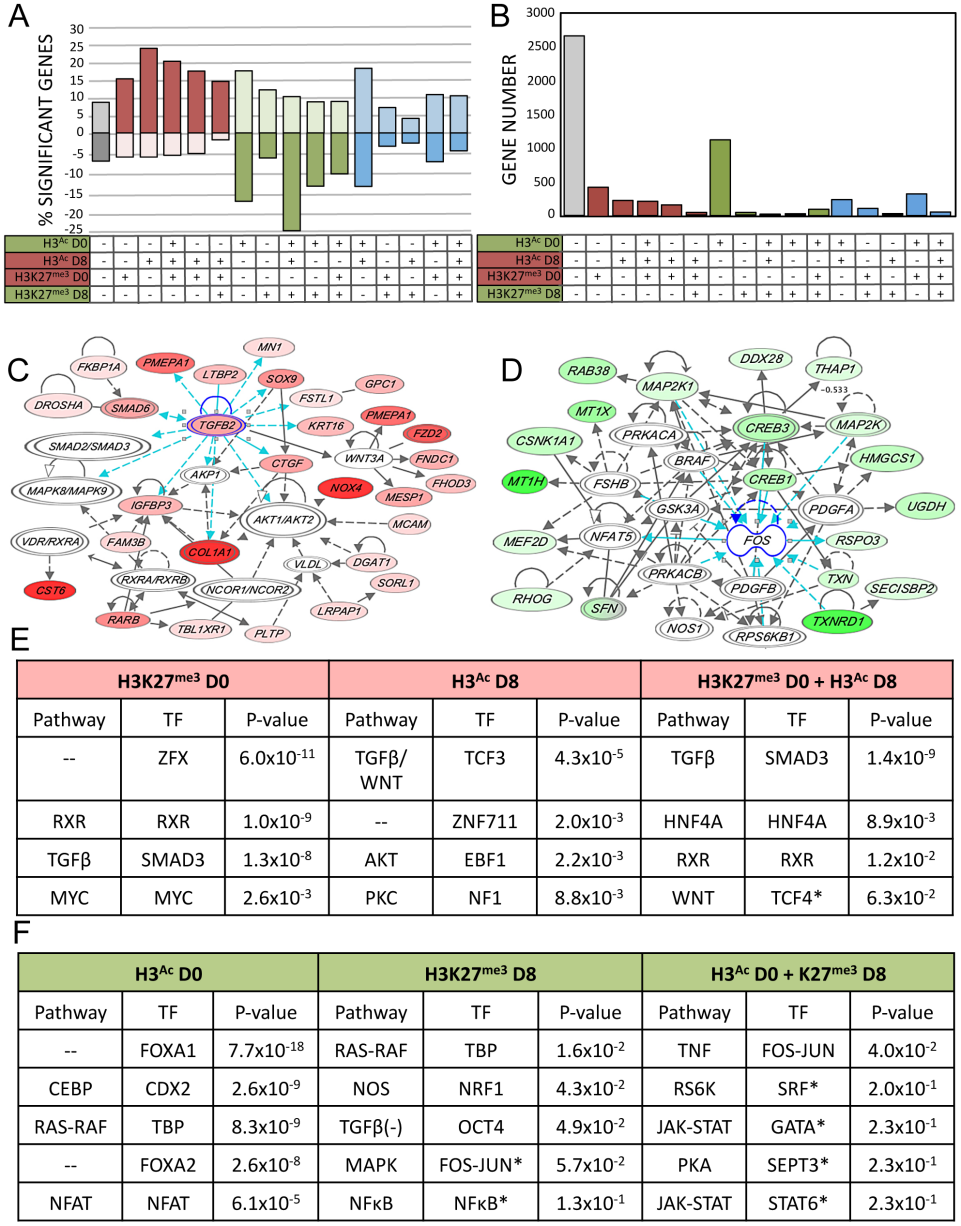
Integration of gene expression data with epigenetic alterations. A–B–C) Relationship between all 16 possible combinations of chromatin changes and gene expression. Grey = unassociated with H3K9/14^Ac^ or H3K27^me3^ changes, red = potentially activating chromatin changes, green = potentially repressive chromatin changes, blue = mixture of both. A) Significant expression changes in genes as a percentage of all genes associated with each histone mark for each of the possible 16 combinations of chromatin marks. Upregulated = above x-axis, downregulated = below x-axis. B) Total number of genes with significant gene expression changes associated with each chromatin combination. C) Representative IPA network of upregulated genes with both H3K9/14^Ac^ gain and H3K27^me3^ loss. D) Representative IPA network of downregulated genes with H3K9/14^Ac^ loss. E and F) IPA ranked networks of genes subset by chromatin context. Corresponding TFBS present in subset chromatin and enrichment p-value from HOMER analysis, for each chromatin-associated gene subset. Red = upregulated gene expression and activating chromatin changes, green = downregulated gene expression and deactivating chromatin changes. (*) Indicates below significance threshold in HOMER but still present in IPA.

Activating chromatin changes were strongly associated with upregulated gene expression, while repressive chromatin changes were loosely associated with downregulated gene expression (Figure 4A). 3011 (53%) of the genes showing altered expression were associated with at least one mark, and the largest single category was that of genes showing only acetylation at D0 (Figure 4B). Since we sampled just two of several dozen known histone tail post-translational modifications, the statistically significantly activated or repressed genes that were not associated with either of these marks may be regulated by epigenetic events and chromatin marks not evaluated in the current study.

We performed IPA analysis on sets of genes associated with genomic regions carrying combinations of active or repressive chromatin marks that were associated with positive and negative expression changes respectively (Figure 4C). We simultaneously performed TFBS motif analysis on those genomic regions. For each class, we investigated the most significant TFBS matches, which were then matched to a transcription factor identified in the corresponding IPA gene networks enriched in the same combined chromatin/expression class (Figure 4C–D). Upregulated pathways included TGFB, WNT, HNF4A, RXR, and AKT (Figure 4E), while downregulated pathways included CEBP, RAS-RAF, and tumor necrosis factor (TNF) as well as FOXA1 and FOXA2 (Figure 4F). This three-way integration of whole-genome gene expression data, chromatin data, and TFBS identified several pathways already implicated in AEC differentiation, such as the WNT and TGFB signaling pathways [35], [36], along with transcription factors previously implicated in lung development such as FOXA1 and FOXA2 [7], [36]. Data integration allowed us to distinguish those genes (and regulatory sequences) likely to be *direct* transcriptional targets for each pathway, such as *PGC*, encoding an AT2-specific protease [37] as a potential direct target of FOXA1 (Figure 3D). Our analysis identified several pathways not previously implicated in lung regeneration, such as RXR, HNF4A, and TNF. The chromatin data enabled us to focus on those genes likely to be directly targeted by these novel pathways, such as *FZD2*, encoding a WNT receptor and candidate target of RXR (Figure 3C).

### Validation of predicted transcription factor signaling pathways

As noted, one signaling pathway previously reported to be involved in AEC differentiation [35], [38], [39] and confirmed through our integrated analysis is the WNT signaling pathway. Specific examination of the microarray data for genes in the WNT signaling pathway showed altered expression of many genes, verified through qRT-PCR (Figure S15) and enrichment of the WNT signaling target transcription factor TCF4 in activated chromatin regions (Figure 3B, Figure 4E).

**Figure 5:**
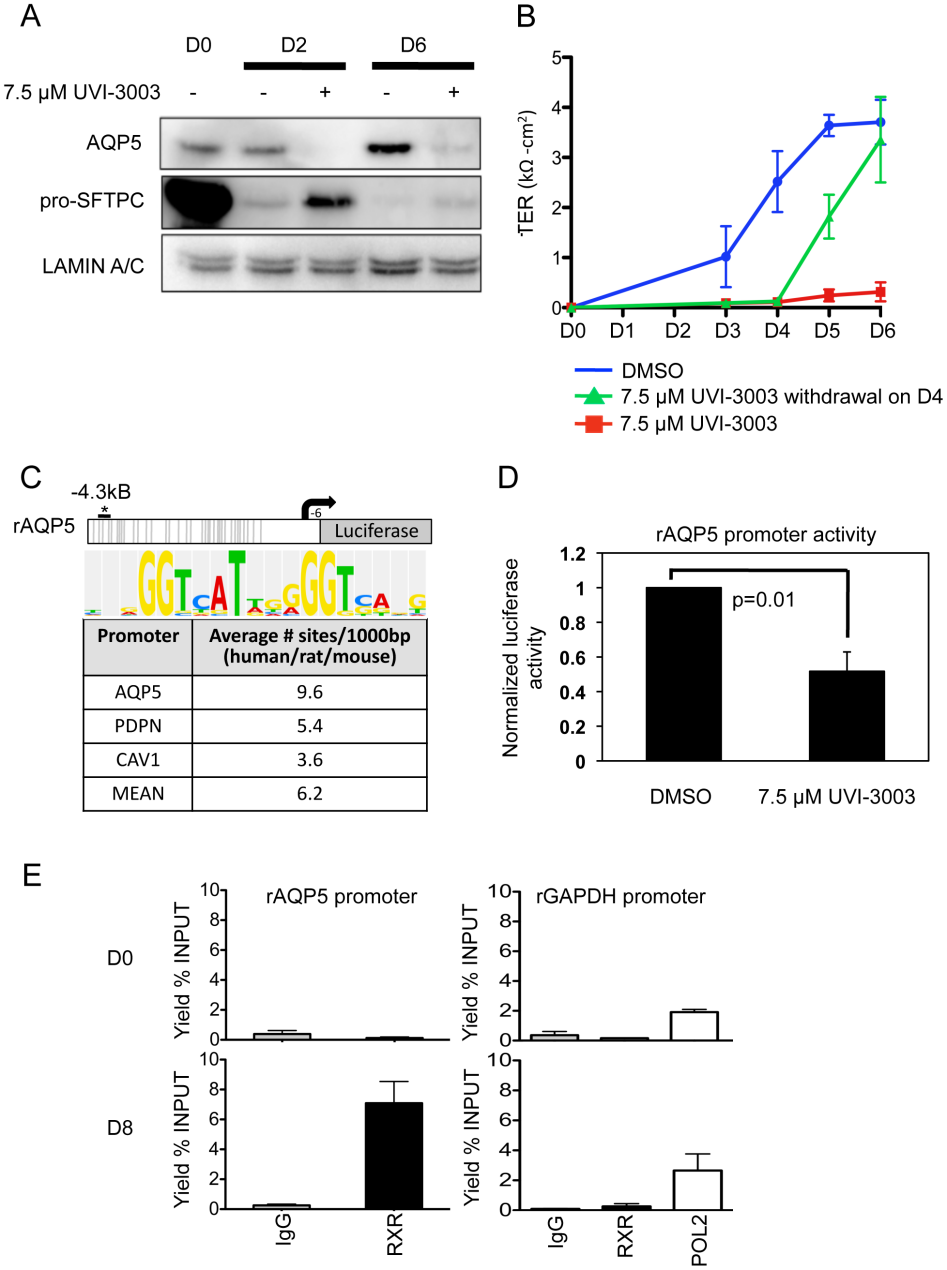
Functional validation of a transcription factor signaling pathway predicted from bioinformatics analysis. A) Western blots examining AT2 and AT1 cell markers during differentiation in the presence or absence of RXR antagonist UVI-3003. LAMIN A/C is the loading control. B) Transepithelial resistance as measured in kΩ-cm^2^ over the course of differentiation. Error bars represent technical duplicates for each plating. C) Rat *Aqp5*-luciferase 4.3 kb promoter construct. Grey lines = 34 putative PPARA:RXR binding sites (Explain3.0). No sites were predicted from −900 to +6 bp due to lack of rat sequence information in the Explain v3.0 database. The asterisk marks the approximate location in the promoter of the ChIPed RXR site in E, below. The average number of PPARA:RXR sites per kilobase in the listed human/rat/mouse promoters is given in the table, with consensus site listed at the top. D) MLE-15 cells were transiently transfected with the *Aqp5*-luciferase construct and treated for 48 hours with vehicle (DMSO) or 7.5 µM UVI-3003. UV1-3003 treatment reduced *Aqp5*-luc activity by 48%±0.06. Values were normalized to vehicle control and represent the mean, error bars represent SEM, N = 3. All experiments represent 3 biological replicates. E) ChIP was performed on primary cultured rat AEC at day 0 (AT2, D0, n = 2) and day 8 (AT1-like, D8, n = 3). A region ∼4 kb upstream of the transcription start site specifically precipitated with RXR in day 8 samples. ChIP of GAPDH with RXR was performed as a control, and POL2 (POLR2A) binding to the GAPDH promoter was included as a positive control for the quality of day 0 DNA.

Of the newly implicated pathways, we chose the RXR pathway for validation. Retinoid X receptors can homodimerize or heterodimerize with a large variety of proteins, including retinoic acid receptors (RARs), thyroid hormone receptor (THR), the vitamin D receptor (VDR), farnesoid X receptor (NR1H4) and other nuclear receptors (NRs), as well as the family of peroxisome proliferator-activated receptors (PPARs) [40]. While retinoic acid and its receptor has been implicated in lung development and injury [41]–[48] the precise role of RXR remains to be clarified. Because of their many potential binding partners, the function of RXRs can be complex; certain NR heterodimers (e.g. NR1H4:RXR, PPAR:RXR) are permissive, responding to an RXR ligand (“rexinoid”) or the corresponding NR ligand, while other complexes are non-permissive, responding only to rexinoids in the presence of ligands for the NR partner (such as RXR:RAR, RXR:VDR, RXR:THR) [49]. Our integrated data suggested RXR-based activation of certain genes that lose the H3K27^me3^ mark (Figure 4E). To test a functional role for RXR pathway activation in AEC differentiation, it was important to target these receptors specifically. Thus, freshly isolated rAT2 cells were plated in the presence or absence of UVI-3003 (7.5 µM), a selective RXR inhibitor that blocks transactivation by RXR agonists but has been previously demonstrated to have minimal binding to structurally similar RAR family members [50]. Treatment with UVI-3003 markedly reduced AT1 cell marker induction (AQP5) while delaying downregulation of AT2 cell marker pro-SFTPC (Figure 5A) and significantly reducing transepithelial resistance (p<0.0001) (Figure 5B). Rescue of UVI-3003 treatment by drug removal on D4 of differentiation restored tight junctions. Analysis of the regulatory region of rat *Aqp5* (a well-established AT1-specific gene) revealed 34 predicted PPARA:RXR heterodimer binding sites (Figure 5C) and an average of 9.6 predicted PPARA:RXR sites per kb across 4 kb upstream regions of the rat, mouse, and human *AQP5* promoters. A similar phenomenon was seen with other AT1-specific genes (*Pdpn, Cav1*) (Figure 5C). Luciferase assays using the 4.3-kb upstream region of the rat *Aqp5* gene transfected into mouse lung epithelial (MLE-15) cells revealed that UVI-3003 inhibited ∼50% of *Aqp5* promoter activity (Figure 5D). ChIP assays of cultured rAEC revealed little detectable binding of RXR to the *Aqp5* promoter at D0, but a marked increase in precipitation of a site about 4 kb upstream of the transcription start site was seen at D8, when the cells had achieved their AT1 cell-like phenotype (Figure 5E). Taken together, these analyses support a function for RXR signaling in AEC differentiation, and illustrate the utility of an integrated transcriptomic/epigenomic approach to identify new pathways involved in differentiation.

## Discussion

AT1-like cells, differentiated *in vitro* from AT2 cells, exhibit many properties of AT1 cells *in vivo*, including morphology and expression of known phenotypic markers. Direct isolation of fragile AT1 cells from human lung is very challenging [51], due in part to the fact that strong cell-specific markers remain to be identified. However, the ability to differentiate AT2 cells *in vitro* into AT1-like cells offers a tractable model system to study not only the transcriptomic and epigenomic differences between these two cell types, but also the kinetic mechanisms controlling epithelial cell differentiation. Transcriptomic analysis of differentiating primary human and rat AEC identified thousands of genes undergoing significantly altered expression, a large number of which overlap between the two species. ChIP-seq identified D0- and D8-specific acetylation (H3K9/14^Ac^) and methylation events (H3K27^me3^) and allowed identification of corresponding TFBSs enriched within AT2- or AT1-specific chromatin patterns. Interestingly, almost all of the TFBS enriched within D8-specific H3K9/14^Ac^ regions were also enriched within D0-specific H3K27^me3^ regions, and conversely for D0-specific H3K9/14^Ac^ and D8-specific H3K27^me3^. This near perfect concordance is unexpected since H3K27^me3^ is thought to represent only one of several silencing mechanisms active in development [3].

Integrated analysis showed that upregulation of gene expression was associated with individual or combined gain of H3K9/14^Ac^ and loss of H3K27^me3^, while downregulation was primarily associated with loss of H3K9/14^Ac^. Approximately half of the genes showing altered expression were not associated with either chromatin mark. This could be because marks were present but distant from the gene, or because other chromatin marks or regulatory mechanisms were involved in their up or downregulation. With application of ChIP-seq to the examination of other chromatin marks, remodeling complexes, and transcription factors, and the addition of information on nucleosome positioning, non-coding RNAs, microRNAs, chromatin conformation capture technologies, and DNA methylation, the current model system will lend itself well to a detailed understanding of the epigenomic basis for the differentiation of adult epithelial cells.

This study demonstrates how expression datasets and chromatin mapping are a potent combination to obtain an integrated picture of signaling pathway activity, transcription factors and their genomic targets. Transcriptional profiling can identify altered gene expression and corresponding regulatory pathways, but identifying transcription factors is difficult without knowing which genomic regions are implicated; epigenomic profiling can pinpoint the specific genomic regions where transcription factors and other regulatory proteins are likely to bind. Our approach revealed known regulators, such as TGFB, WNT, FOXA1 and FOXA2 [52]–[55], [7] as well as new potential regulators of AEC differentiation, including AKT, RXR and HNF4A.

As proof-of-principle, we investigated whether RXR signaling was required for the differentiation process of adult alveolar epithelium. Use of an RXR-specific inhibitor delayed differentiation as measured by inhibition of AT1-specific AQP5 expression and delayed TER. A role for RXR in normal AQP5 expression in AT1 cells is further supported by the inhibition of the *Aqp5* promoter by UVI-3003, as well as the specific detection of RXR on the *Aqp5* promoter in rAT1-like cells. Retinoic acid receptors (RARs) are one of the many potential binding partners with RXRs and numerous reports have implicated retinoic acid and/or RARs in lung development and injury [40]–[49]. Thus, it is possible that RXR effects are mediated through interactions with RAR. However, evaluation of stringently predicted RXR binding sites in the AQP5 promoter in human and rat shows that the presence of adjacent predicted RAR binding sites is rare or absent, while adjacent predicted VDR sites are more common, and predicted estrogen and glucocorticoid receptor sites also abut RXR binding site [56]. Given the multitude of interacting partners of RXRs (over 19 described [40]), dissecting the mechanism of RXR action on the promoter of AQP5 and other genes will require a very detailed examination. While the molecular mechanisms of altered RXR signaling remain to be further defined, inhibition of RXR *in vitro* as well as ChIP data showing increased promoter occupancy in AT1-like cells on day 8 support its functional role and illustrate the utility of our system and the potential of epigenomic/transcriptomic data integration to reveal novel regulators of biologic processes.

In summary, our analysis enabled identification of known and novel signaling pathways, gene regulatory networks and associated TFBS implicated in morphologic and phenotypic changes that occur during AEC differentiation. Full characterization of normal differentiation is critical to determine the precise mechanisms that are perturbed in disease. In the distal lung, this might shed light on the molecular basis of chronic obstructive pulmonary disease and idiopathic pulmonary fibrosis. The analysis presented here shows that purified human primary epithelial cells undergoing *in vitro* differentiation can serve as a powerful tool for the mechanistic investigation of normal and aberrant epithelial cell differentiation.

## Materials and Methods

### Ethics statement

Remnant human transplant lungs were obtained in compliance with Institutional Review Board-approved protocols for the use of human source material in research (HS-07-00660) and processed within 3 days of death. Rat AT2 cells were isolated in compliance with IACUC protocol #11360.

### Isolation and culture of human and rat alveolar epithelial cells

Human lung tissue was processed as previously described [29] with inclusion of anti-EpCAM conjugated beads to select for epithelial cells. Cells were plated in 50∶50 [DMEM High glucose media (GIBCO 21063): DMEM-F12 (Sigma D6421)]. Differentiation into AT1-like cells was verified by measuring *SFTPC* and *AQP5* expression using RNA extracted with the Illustra TriplePrep Kit (GE LifeSciences, Piscataway, NJ), and by measuring transepithelial resistance. Rat AT2 cells were isolated as previously described [57], [19]. Total RNA, DNA, and protein were simultaneously isolated from AT2 cells (D0), intermediate cell phenotypes (D2-6) and AT1-like cells (D8). Chromatin was isolated in tandem at D0 and D8.

### Immunofluorescence

Freshly isolated hAT2 cells were fixed with 4% paraformadehyde for 10 min at room temperature (RT), permeabilized with 0.3% Triton, and blocked with CAS blocking reagent (Invitrogen Cat #00-8020, Camarillo, CA) for 30 min at RT. Slides were incubated with rabbit anti-pro-SFTPC (Seven Hills #WRAB-SPC serum) or anti-PTPRC (Santa Cruz sc-25590) antibodies and diluted in CAS-block at 4°C overnight. Slides were washed in Tris-Buffered Saline & Tween 20 (TBST) and incubated with anti-rabbit-FITC fluorescent secondary antibody in CAS-block for 1 hr at RT. For vimentin staining, mouse anti-VIM (Sigma V2258) and biotinylated anti-mouse IgM (Vector # BA-2020) antibodies were used. Sections were viewed with a NIKON Eclipse microscope equipped with a QImaging Retica 200R charge-coupled-device camera (QImaging, Surrey, BC, Canada). Images were processed with the NIS-Elements BR program (NIKON).

### Gene expression analysis by Illumina HT-12v4 and RatRef-12 arrays

1 µg of RNA was converted into cRNA using Illumina TotalPrep RNA amplification kit, (Life Technologies, USA) and used for Illumina HT-12v4 or RatRef-12 expression analysis at the Southern California Genotyping Consortium, University of California Los Angeles. BeadStudio was used to convert images to raw signal data. Using R (version 2.11.1), Variant Stabilization and Normalization (VSN) was performed using LUMI [58] to allow for a large number of differentially expressed genes. Statistical analyses were performed using LIMMA [59]. A linear regression model was fitted over the time-course of differentiation, technical replicates were removed, and t-tests performed between D0 and D8. False-discovery rate was controlled using the Benjamini-Hochberg (BH) correction [60]. R was used for principal component analysis and heatmap generation. Heatmaps were generated by selecting the top 5% of probes most variant across the whole dataset and clustering with Ward's method. Pathways analysis was performed using IPA (Ingenuity Systems, www.ingenuity.com) or DAVID [61], [62]. Correlation of human and rat gene expression was performed using Entrez identifiers and the Mouse Genome Informatics (MGI) Web database [63].

### Quantitative polymerase chain reactions

RNA was reverse transcribed using random hexamers and M-MLV reverse transcriptase per manufacturer's guidelines (Invitrogen), followed by qRT-PCR using SYBR green (BioRad, Hercules, CA) with primers listed in Table S2. qRT-PCR reactions were performed using a DNA engine Opticon (MJ Research, Waltham, MA) and normalized to 18S rRNA.

### ChIP and ChIP-seq on primary human and rat epithelial cells

Chromatin immunoprecipitations for H3K9/14^Ac^ (Millipore, #06-599), H3K27^me3^ (Millipore, #07-449), POL2 (POLR2A, Santa Cruz Biotechnology, sc-899X), and RXR (Santa Cruz Biotechnology, sc-774X) were performed as previously described [64]. Primer sequences are noted in Table S3. For rat ChIP at the *Gapdh* promoter, POL2 was used as a positive control. For human ChIP-seq, positive control loci were the promoter regions of *GAPDH* and *MUC4* for H3K9/14^Ac^ and H3K27^me3^ respectively; ≥10-fold enrichment was considered successful. ChIP-seq libraries were constructed using the New England Biolabs library prep kit (NEB Cat#E6200, Ipswich, MA). ChIP products underwent Illumina GAII single-end sequencing; reads were aligned to the hg18 human genome build using the MAQ 0.7.1 aligner. All datasets are deposited in the public GEO database (GEO# GSE38571).

### Data access

All datasets are deposited in the public GEO database (GEO# GSE38571).

### Peak calling

Integrative Genomics Viewer v1.5 (The Broad Institute) was used to visually inspect peak quality. SICER [65] peak calling was performed using a window and gap size of 600 bp. Input DNA was used for background normalization. The second peak-calling algorithm, MACS, (http://cistrome.org/ap/) was performed using default parameters and input DNA for background normalization. SICER- and MACS-called peaks had a large degree of overlap, as measured by the correlation coefficient (Figure S16A), calculated using *Genome Graphs* (http:/genome.ucsc.edu). A lack of correlation was observed between H3K9/14^Ac^ and H3K27^me3^ (Figure S16B) [66]–[70]. ChIP-seq reads from both AT2 and AT1 cells were observed at or near read saturation (Figure S17). Differential peak occupancy was determined using the UCSC table browser. Manhattan plots were generated using *Genome Graphs*.

### Motif analysis & data integration

Peaks were annotated to the nearest transcription start site (TSS) and motif analysis was performed with HOMER [34]. The opposite AEC cell type was used as background. Gene expression data was merged with annotated chromatin peaks using Entrez ID. For the AQP5 TFBS analysis PPAR:RXR predicted binding sites were evaluated using P-match algorithms within ExPlain 3.0 across human, mouse and rat species. ExPlain motif V$PPARA_02 with a high probability (>87%) were counted and averaged in the region 4.3 kb upstream of the promoter for the three species. Similar results were obtained with the Match program in Biobase [56], which was used to examine the frequency and nature of binding site adjacent to predicted RXR targets.

### RXR inhibitor studies

1×10^6^ rat AT2 (≥95% purity) cells were plated on 1.1-cm^2^ polycarbonate filters (Corning Costar #3401) and treated with either 7.5 µM UVI-3003 (Tocris Biosciences, Ellisville Missouri) or DMSO vehicle control from the time of plating (D0) through completion of the study. Whole cell lysates were extracted in 2% SDS lysis buffer (62.5 mM Tris pH 6.8, 2% SDS, 10% glycerol, and protease inhibitor cocktail III (Calbiochem #539134)).

### Western blot analysis

Western blots were performed as previously described [16]. Primary antibodies (all rabbit) were anti-AQP5 (Alomone Labs AQP-005), anti-CAV1 (Abcam ab2910), anti-pro-SFTPC (Millipore AB3786) and anti-LAMIN A/C (sc-20681, Santa Cruz Biotechnology). Blots were analyzed by chemiluminescence and visualized by West Fempto Super Sensitivity Kit (Thermo Scientific) with a FluorChem 8900 Imaging System (Alpha Innotech).

### Luciferase assays

Mouse lung epithelial cells (MLE-15) were plated at a density of 4×10^4^ cells per well on 24-well plates 1.5 days prior to transfection. Duplicate wells were transiently transfected with 500 ng r*Aqp5*-luc containing the 4.3 kb rat *Aqp5* promoter or pGL2 empty vector using Superfect (Qiagen). Three hours post-transfection, media was changed to contain 7.5 µM UVI-3003 or DMSO vehicle control. 48 hours post transfection, luciferase assays were performed as previously described [33]. Raw luciferase values were normalized to protein concentration and then to pGL2 empty vector controls. Significance was measured using the student's t-test.

## Supporting Information

Figure S1 Quality control for human AT2 cell extraction and differentiation. A) Information on the three subjects from whom AEC were obtained, showing the color-coding used in Figure 1. B) Cytocentrifuged preparations of freshly isolated human AT2 cells were stained with the indicated antibodies (green). PTPRC = hematopoietic cell marker, VIM = vimentin, a mesenchymal cell marker, SFTPC = AT2 cell marker. Propidium iodide (PI) was used for nuclear counterstaining (red). C) RNA isolated from differentiating AT2 cells at the indicated time points was subjected to qRT-PCR. AQP5 = aquaporin 5, AT1 cell marker. D) TER (measured in kΩ-cm^2^) for all 3 donor lungs. E) AT1-like hAEC differentiated and stained for tight junction protein. PI = propidium iodide, DNA stain (blue), TJP1 = tight junction protein 1 (orange).
(TIF)

Figure S2 Raw human expression profiling. A) Density plot of raw sample distributions. B) Boxplot of raw sample intensity distributions. C) Raw ranked mean standard deviation of signal for all samples. D) Dendrogram of sample similarity based on top variant genes, those with standard deviation/mean >0.1. E) Distribution of raw p-values.
(TIF)

Figure S3 Preprocessed and normalized human expression profiling. A) Density plot of VSN background corrected and normalized sample distributions. B) Boxplot of VSN-corrected sample intensity distribution. C) Normalized ranked mean standard deviation of signal for all samples. D) Dendrogram of sample similarity based on top variant genes, those with standard deviation/mean >0.1. E) Principal component analysis of normalized sample data. Each bar represents a source of variation. Bar height indicates amount of variation. F) Distribution of p-values for normalized data (x-axis) and their rate of occurrence (y-axis).
(TIF)

Figure S4 Stability of human gene expression clusters. A) Heatmap clustering of top 2% of variant genes between samples, clustering using Ward's method. B) Heatmap clustering of top 10% of variant genes between samples, clustering using Ward's method. C) Silhouette plot using Euclidean distances. Clusters were defined from Heatmap at major branch points as D0 (cluster 1), D2 (cluster 2), and D4, 6, 8 (cluster 3).
(TIFF)

Figure S5 qRT-PCR of top up- and down-regulated genes. Graphs represent relative transcript abundance at each time point during differentiation. Fold change is expressed relative to transcript abundance at D0 (AT2 cells). 18S rRNA served as normalization control. Error bars represent standard error of the mean (SEM) from biological triplicates.
(TIF)

Figure S6 IPA up- and down-regulated networks of human gene expression data. A) IPA upregulated networks. Intensity of red color indicates degree of upregulation, dark red = highly upregulated, light red = modestly upregulated. Blue lines highlight AKT connections. B) IPA downregulated networks. Intensity of green color indicates degree of downregulation, dark green = heavily downregulated, light green = modestly downregulated. Blue lines highlight FOS (AP1) connections.
(TIF)

Figure S7 DAVID pathways analysis of human gene expression data. Separation of the 3000 upregulated genes with greatest calculated significance (red bars) and all significantly downregulated genes (green bars). Data bars expressed as −log_10_ BH-corrected p-value of significance for enrichment as compared to random sampling of the reference set of genes.
(TIF)

Figure S8 Raw rat expression profiling. A) Density plot of raw sample distributions. B) Boxplot of raw sample intensity distributions. C) Raw ranked mean standard deviation of signal for all samples. D) Dendrogram of sample similarity based on top variant genes, those with standard deviation/mean >0.1. E) Distribution of raw p-values. F) Principal component analysis of sample distribution. Color indicates lung preparation by rat.
(TIF)

Figure S9 Preprocessed and normalized rat expression profiling. A) Density plot of VSN background-corrected and normalized sample distributions. B) Boxplot of VSN-corrected sample intensity distribution. C) Ranked mean standard deviation of signal for all normalized samples. D) Dendrogram of sample similarity based on top variant genes, those with standard deviation/mean >0.1. E) Distribution of p-values from normalized data (x-axis) and their rate of occurrence (y-axis). F) Principal component analysis of normalized sample data. Each bar represents a source of variation. Bar height indicates amount of variation.
(TIF)

Figure S10 Stability of rat gene expression clusters. A) Heatmap clustering of top 20% of variant genes between samples, clustering using Ward's method. B) Heatmap clustering of top 5% of variant genes between samples, clustering using Ward's method.
(TIFF)

Figure S11 IPA network analysis of overlapping human and rat significant gene expression changes. A) IPA (left) and DAVID (right) of genes with significantly altered expression in both human and rat. Data bars expressed as −log_10_ of the BH-corrected p-value for enrichment of each pathway as compared to background. B) Top IPA network analysis of overlapping significant genes in human and rat expression. Red = upregulated in AT2 to AT1 cell differentiation, green = downregulated in AT2 to AT1 cell differentiation. Intensity of color is indicative of degree of fold change in expression.
(TIF)

Figure S12 IPA analysis of human-specific gene expression changes. A) IPA (left) and DAVID (right) of human-specific significant gene expression. Data bars expressed as −log_10_ of the BH-corrected p-value for enrichment of each pathway as compared to background. B) Top IPA network of human-specific changes. Red = upregulated in AT2 to AT1 cell differentiation, green = downregulated in AT2 to AT1 cell differentiation. Intensity of color is indicative of degree of fold change in expression. Blue lines highlight *HNF4A* connections.
(TIF)

Figure S13 IPA analysis of rat-specific gene expression changes. A) IPA (left) and DAVID (right) of rat-specific significant gene expression. Data bars expressed as −log_10_ of the BH-corrected p-value for enrichment of each pathway as compared to background. B) Top IPA network of rat-specific changes. Red = upregulated in AT2 to AT1 cell differentiation, green = downregulated in AT2 to AT1 cell differentiation. Intensity of color is indicative of degree of fold change in expression. Blue lines highlight *Tgfb1* connections.
(TIF)

Figure S14 SICER and MACS called peaks and associated predicted TFBS using genomic background. A) SICER and MACS peaks called for H3K19/14^Ac^ chromatin mark. (Middle) H3K9/14^Ac^ peak overlap between chromatin from D0 and D8 (Venn diagram intersection). Left panel: D0-specific chromatin peaks, right panel: D8-specific chromatin peaks B) SICER and MACS peaks called for H3K27^me3^ chromatin mark. Peak overlap between chromatin from D0 and D8 is at Venn diagram intersection. Left panel: D0-specific chromatin peaks; right panel: D8-specific peaks. For both H3K9/14^Ac^ and H3K27^me3^, chromatin-enriched DNA motifs are listed and the corresponding transcription factors which bind the recognition sequences in an AT2 (left panels) or AT1 cell-specific fashion (right panels).
(TIF)

Figure S15 WNT signaling activated in AT2 to AT1 cell differentiation. A) Canonical WNT signaling pathway. Red = upregulated gene expression, green = downregulated gene expression. B) qRT-PCR of select WNT pathway members over course of AT2 to AT1 cell differentiation. 18S rRNA was used for normalization. Error expressed as SEM, n = 3 separate lung donors.
(TIF)

Figure S16 Summary of ChIP-seq data. A) Summary of ChIPs, sequencing data, and peaks called. IP = immunoprecipitation target. R = correlation coefficient. R squared (R^2^) = degree of correlation. Blue = H3K9/14^Ac^, red = H3K27^me3^, purple = input. B) Table demonstrating the degree of correlation between chromatin states between cell types and between differing histone marks. Blue = H3K9/14^Ac^, red = H3K27^me3^.
(TIF)

Figure S17 Read count has minimal effect on H3K9/14^Ac^ ChIP-seq peak calling. A) SICER peak calling of H3K9/14^Ac^ in AT2 cells as a function of randomly chosen reads from parent H3^Ac^ AT2 read file. B) SICER peak calling of H3K9/14^Ac^ in AT1 cells as a function of randomly chosen reads from parent H3^Ac^ AT1 read file. C) Merged overlap of peaks called as a function of number of reads included in H3K9/14^Ac^ for both AT2 cells (dark blue) and AT1 cells (light blue).
(TIF)

Table S1 HOMER motif analysis of cell-specific TFBS. Each transcription factor (as recorded in HOMER known motifs database) and the associated p-value of enrichment for each day-specific chromatin mark.
(XLS)

Table S2 qRT-PCR primers used in this study.
(DOC)

Table S3 ChIP primers.
(DOC)
