# Supplementary figures and images for: Integrated Transcriptomic and Epigenomic Analysis of Primary Human Lung Epithelial Cell Differentiation
Source: PLoS Genet. 2013 Jun 20;9(6):e1003513. doi: 10.1371/journal.pgen.1003513 (PMC3688557; doi:10.1371/journal.pgen.1003513)

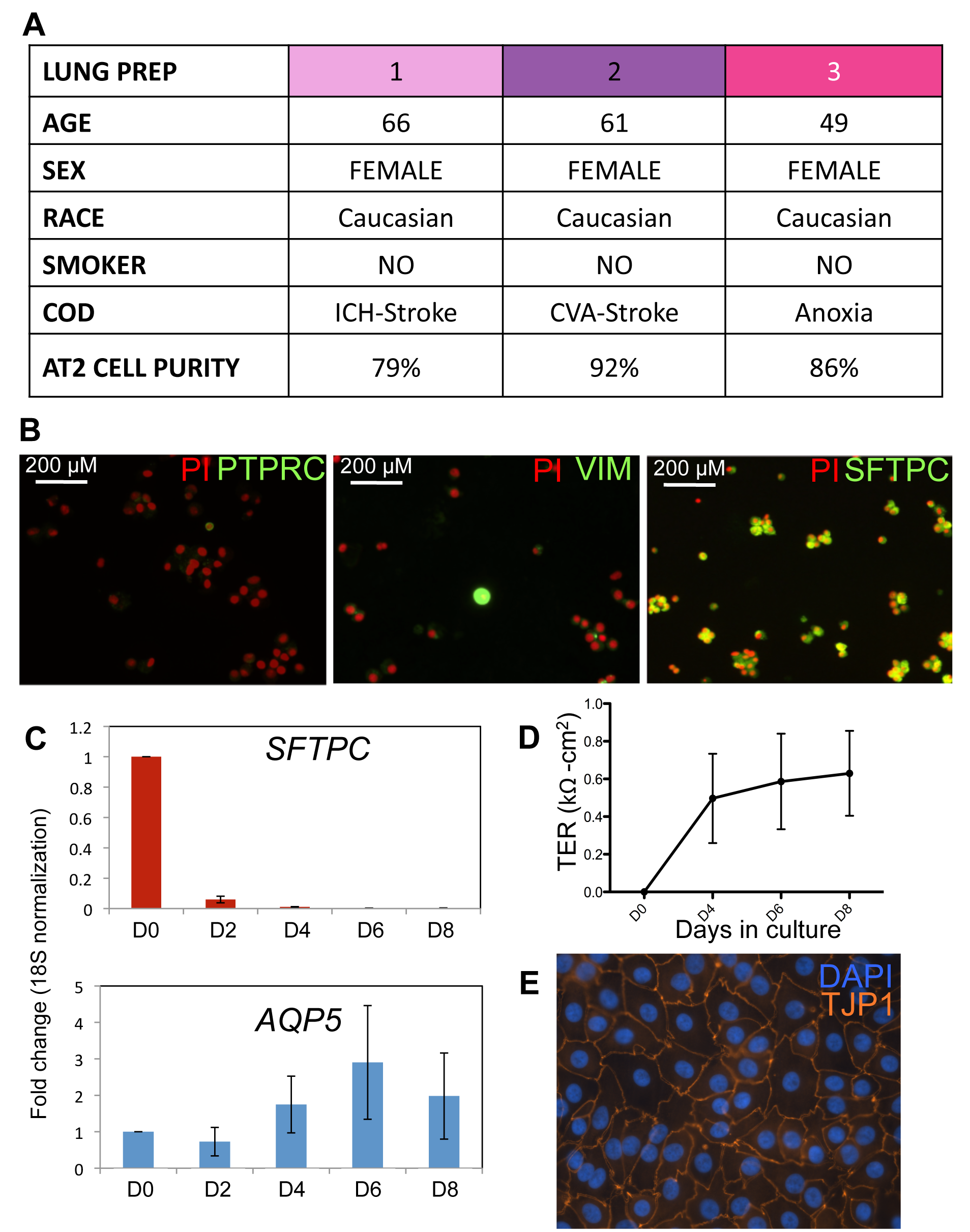

Supplement: Figure S1 — Quality control for human AT2 cell extraction and differentiation. A) Information on the three subjects from whom AEC were obtained, showing the color-coding used in Figure 1. B) Cytocentrifuged preparations of freshly isolated human AT2 cells were stained with the indicated antibodies (green). PTPRC = hematopoietic cell marker, VIM = vimentin, a mesenchymal cell marker, SFTPC = AT2 cell marker. Propidium iodide (PI) was used for nuclear counterstaining (red). C) RNA isolated from differentiating AT2 cells at the indicated time points was subjected to qRT-PCR. AQP5 = aquaporin 5, AT1 cell marker. D) TER (measured in kΩ-cm2) for all 3 donor lungs. E) AT1-like hAEC differentiated and stained for tight junction protein. PI = propidium iodide, DNA stain (blue), TJP1 = tight junction protein 1 (orange). (TIF) [file pgen.1003513.s001.tif]

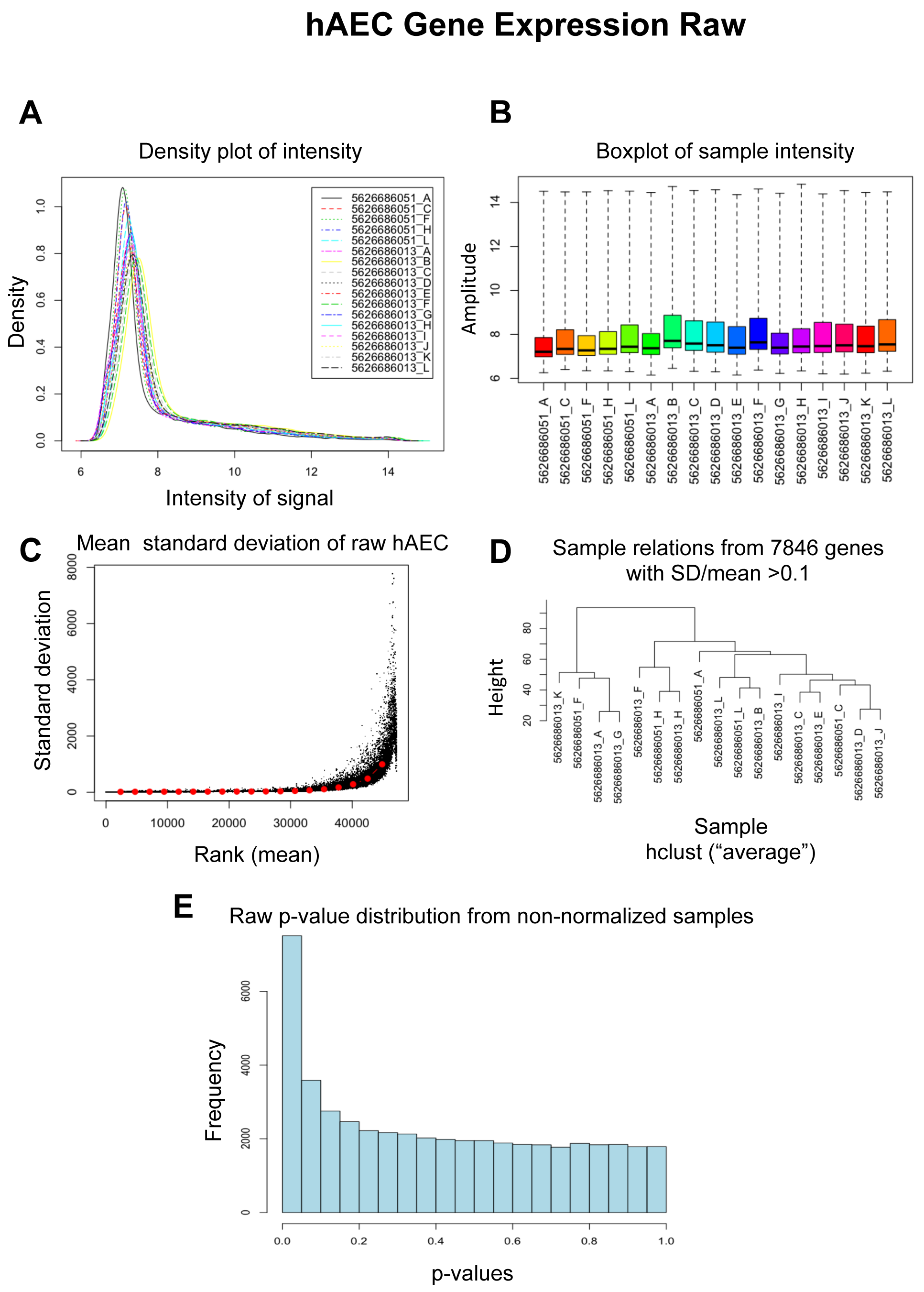

Supplement: Figure S2 — Raw human expression profiling. A) Density plot of raw sample distributions. B) Boxplot of raw sample intensity distributions. C) Raw ranked mean standard deviation of signal for all samples. D) Dendrogram of sample similarity based on top variant genes, those with standard deviation/mean >0.1. E) Distribution of raw p-values. (TIF) [file pgen.1003513.s002.tif]

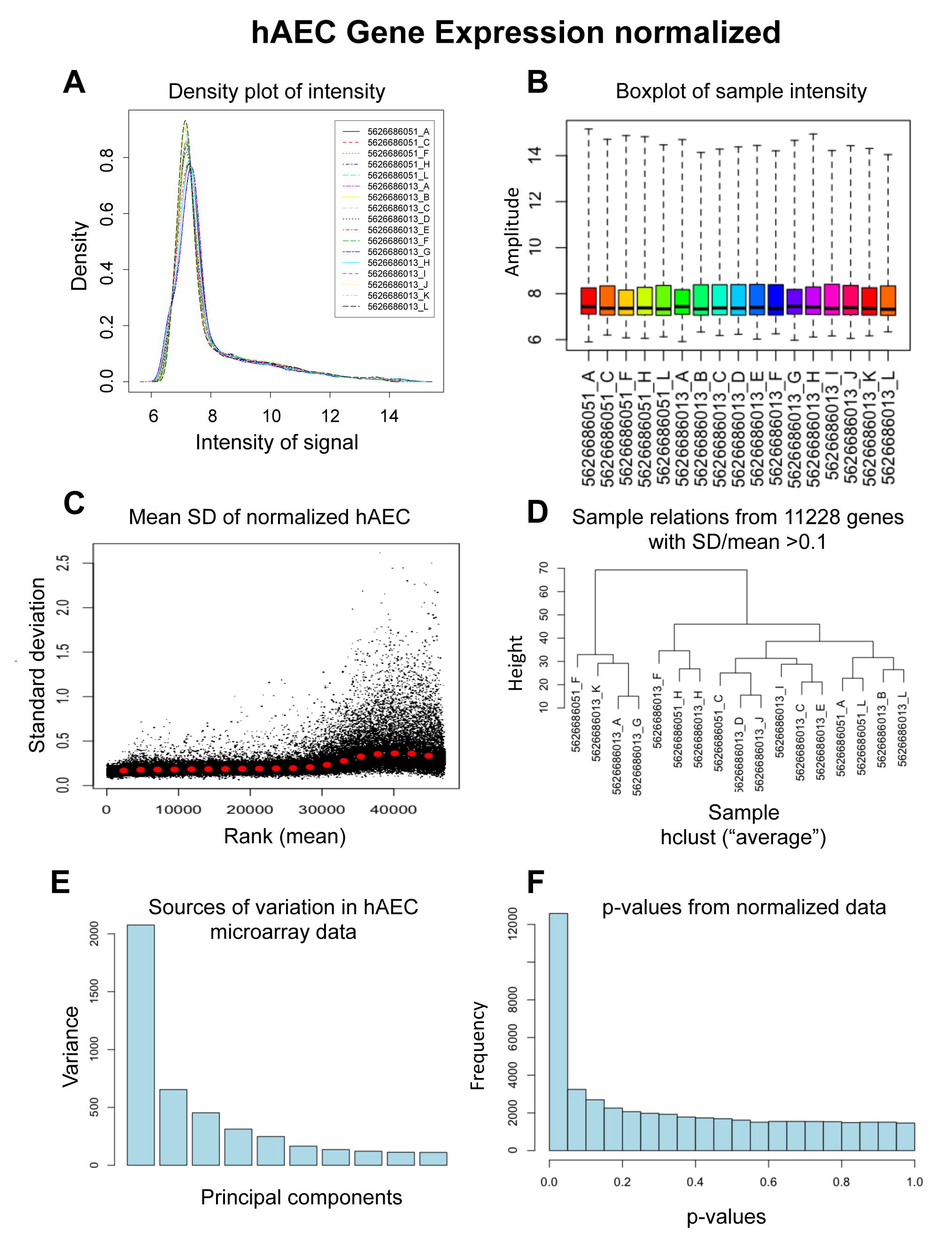

Supplement: Figure S3 — Preprocessed and normalized human expression profiling. A) Density plot of VSN background corrected and normalized sample distributions. B) Boxplot of VSN-corrected sample intensity distribution. C) Normalized ranked mean standard deviation of signal for all samples. D) Dendrogram of sample similarity based on top variant genes, those with standard deviation/mean >0.1. E) Principal component analysis of normalized sample data. Each bar represents a source of variation. Bar height indicates amount of variation. F) Distribution of p-values for normalized data (x-axis) and their rate of occurrence (y-axis). (TIF) [file pgen.1003513.s003.tif]

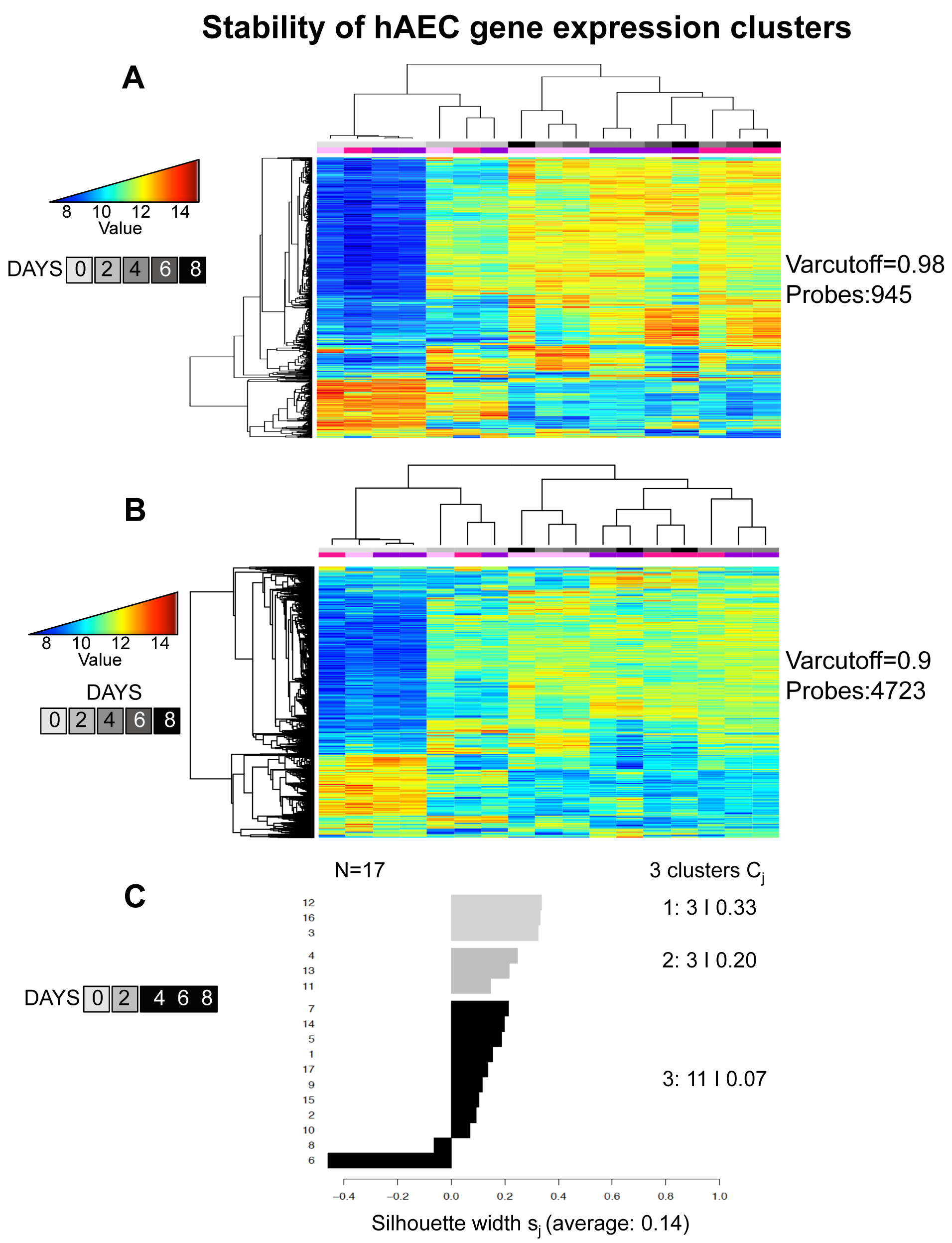

Supplement: Figure S4 — Stability of human gene expression clusters. A) Heatmap clustering of top 2% of variant genes between samples, clustering using Ward's method. B) Heatmap clustering of top 10% of variant genes between samples, clustering using Ward's method. C) Silhouette plot using Euclidean distances. Clusters were defined from Heatmap at major branch points as D0 (cluster 1), D2 (cluster 2), and D4, 6, 8 (cluster 3). (TIFF) [file pgen.1003513.s004.tiff]

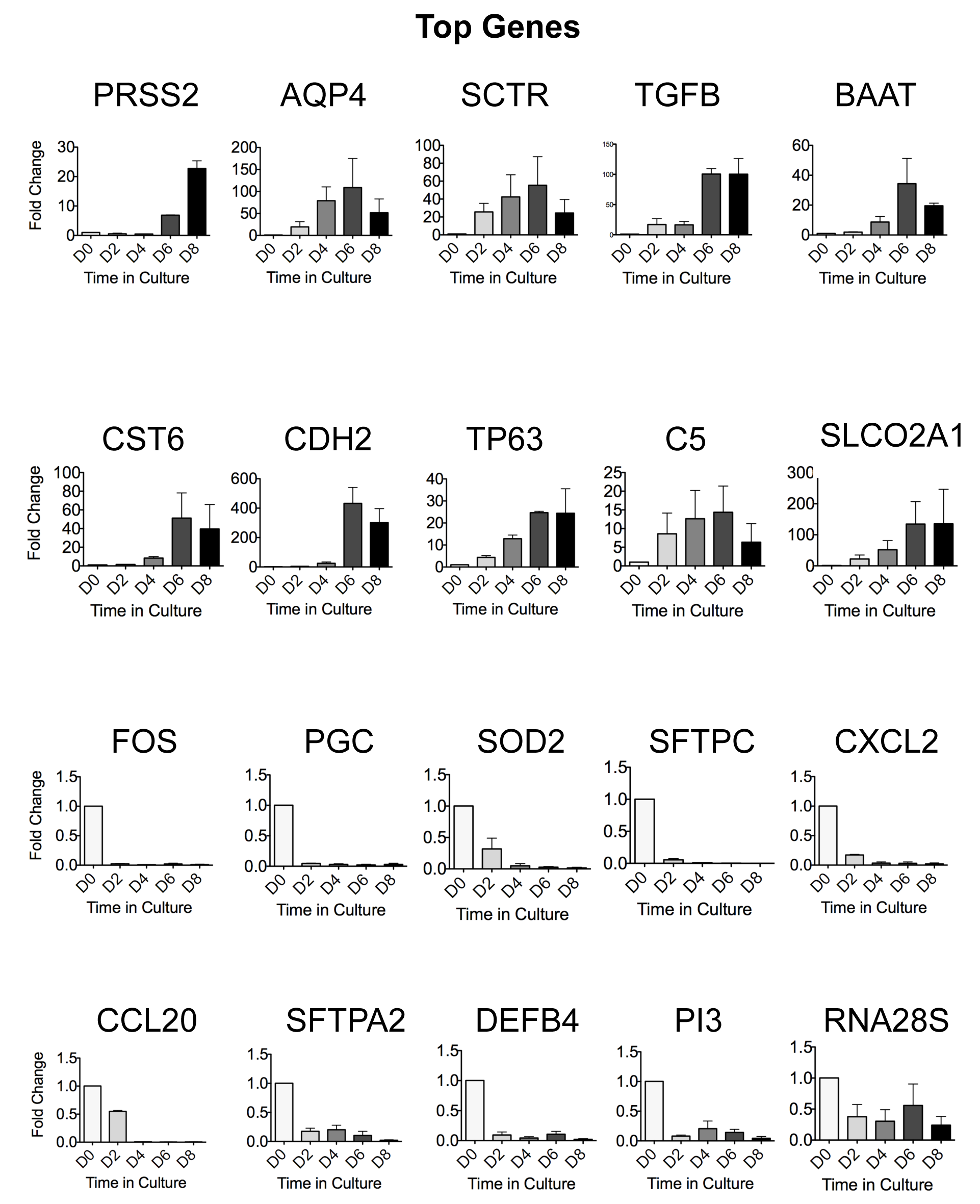

Supplement: Figure S5 — qRT-PCR of top up- and down-regulated genes. Graphs represent relative transcript abundance at each time point during differentiation. Fold change is expressed relative to transcript abundance at D0 (AT2 cells). 18S rRNA served as normalization control. Error bars represent standard error of the mean (SEM) from biological triplicates. (TIF) [file pgen.1003513.s005.tif]

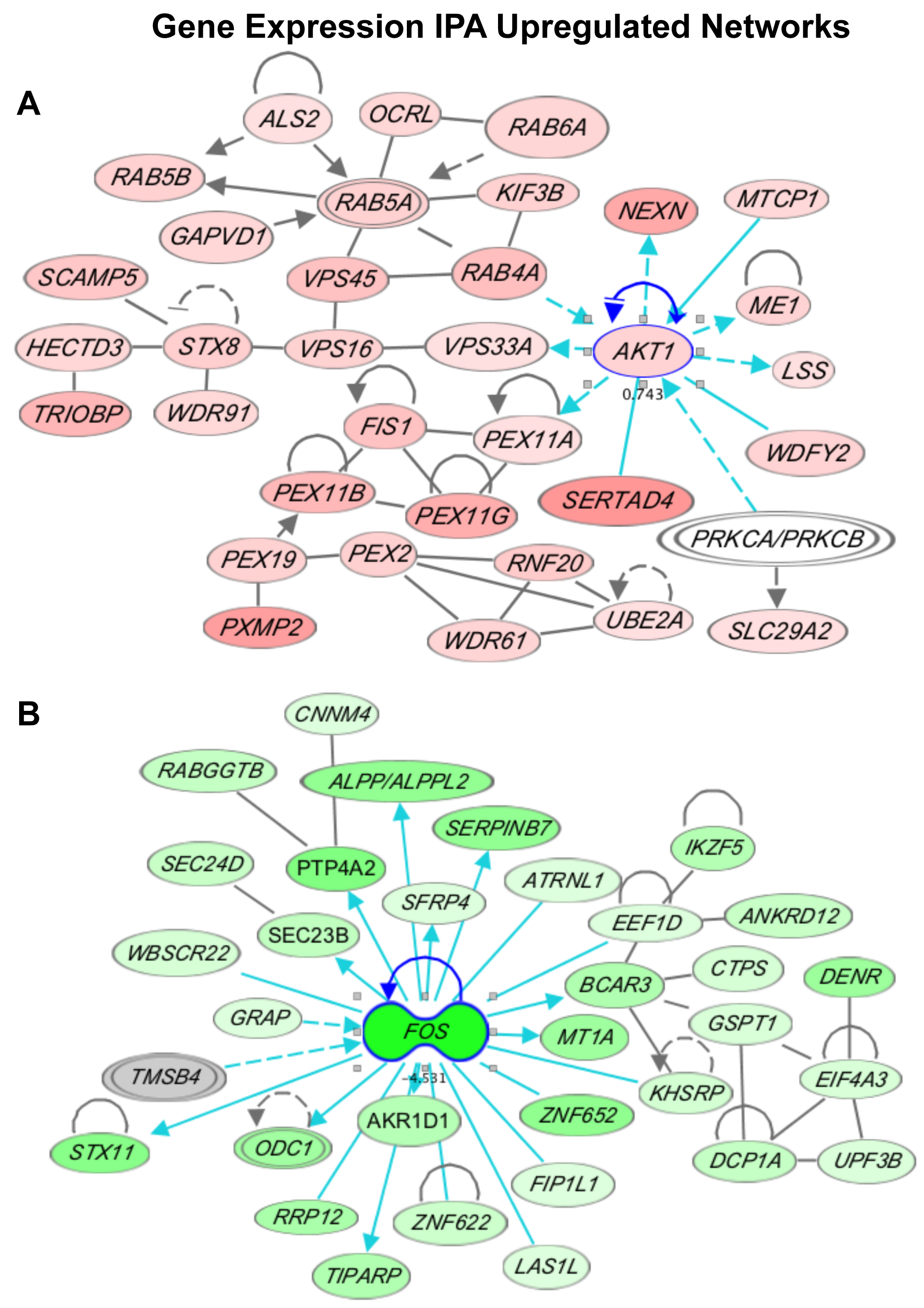

Supplement: Figure S6 — IPA up- and down-regulated networks of human gene expression data. A) IPA upregulated networks. Intensity of red color indicates degree of upregulation, dark red = highly upregulated, light red = modestly upregulated. Blue lines highlight AKT connections. B) IPA downregulated networks. Intensity of green color indicates degree of downregulation, dark green = heavily downregulated, light green = modestly downregulated. Blue lines highlight FOS (AP1) connections. (TIF) [file pgen.1003513.s006.tif]

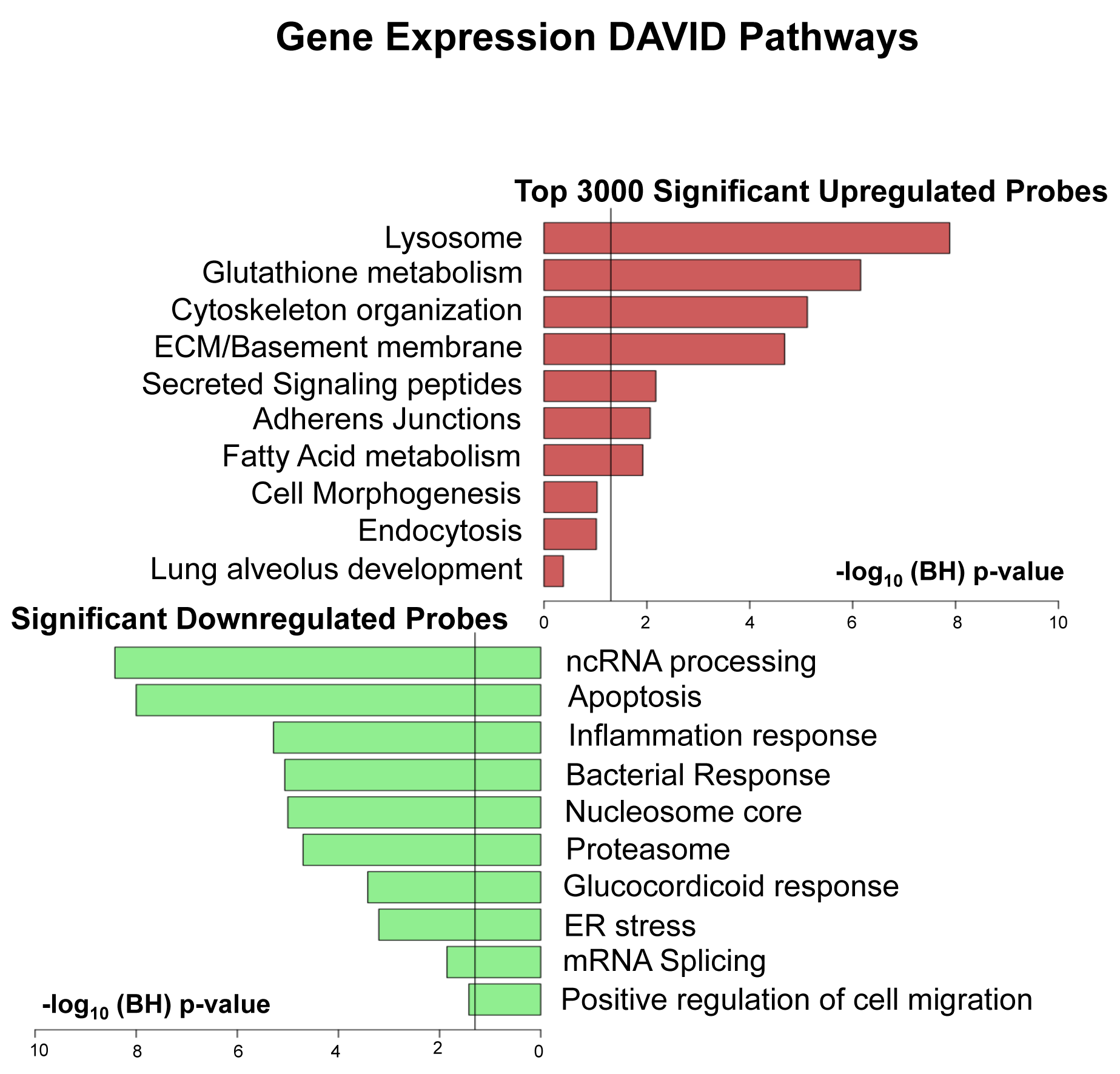

Supplement: Figure S7 — DAVID pathways analysis of human gene expression data. Separation of the 3000 upregulated genes with greatest calculated significance (red bars) and all significantly downregulated genes (green bars). Data bars expressed as −log10 BH-corrected p-value of significance for enrichment as compared to random sampling of the reference set of genes. (TIF) [file pgen.1003513.s007.tif]

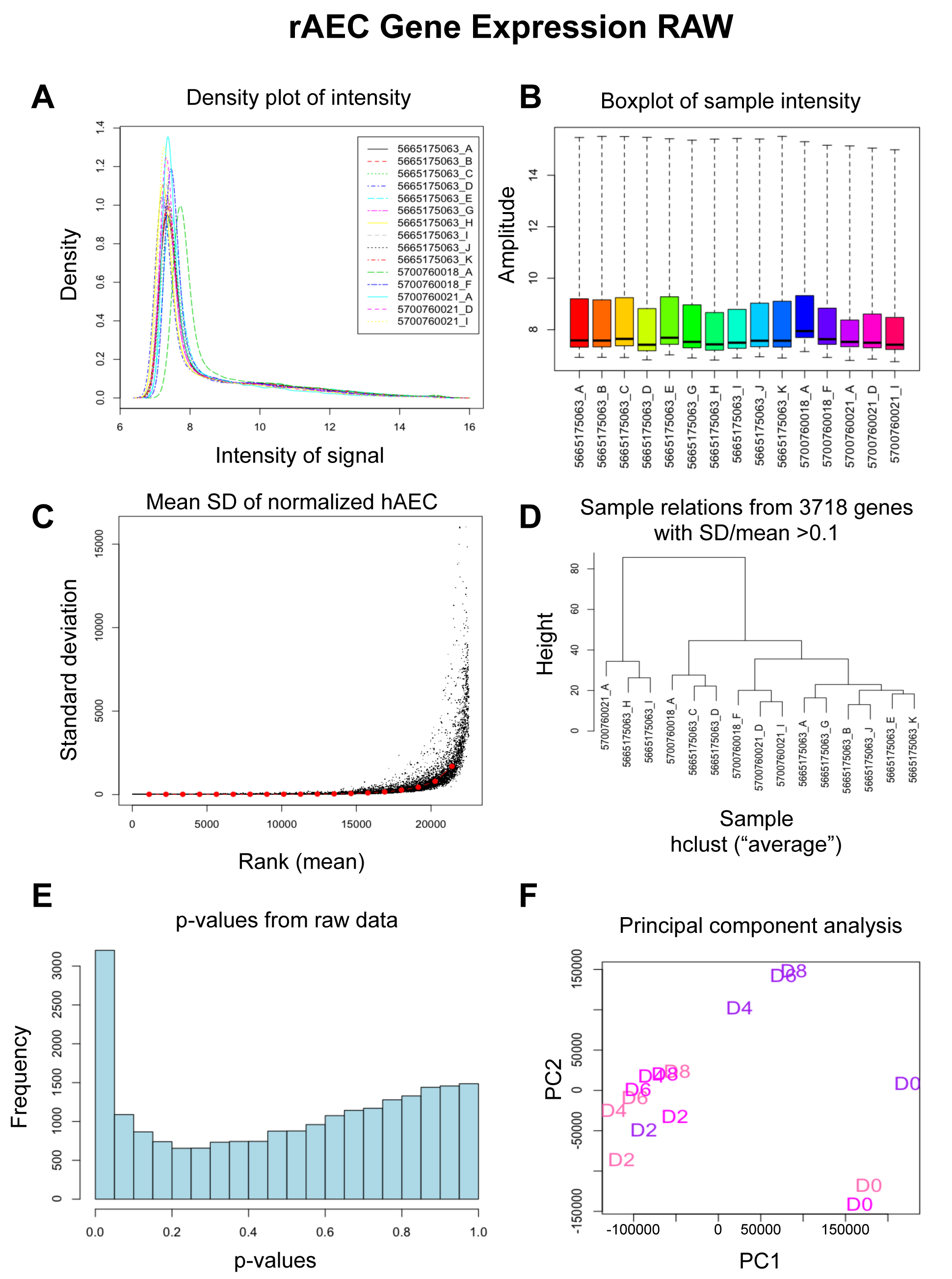

Supplement: Figure S8 — Raw rat expression profiling. A) Density plot of raw sample distributions. B) Boxplot of raw sample intensity distributions. C) Raw ranked mean standard deviation of signal for all samples. D) Dendrogram of sample similarity based on top variant genes, those with standard deviation/mean >0.1. E) Distribution of raw p-values. F) Principal component analysis of sample distribution. Color indicates lung preparation by rat. (TIF) [file pgen.1003513.s008.tif]

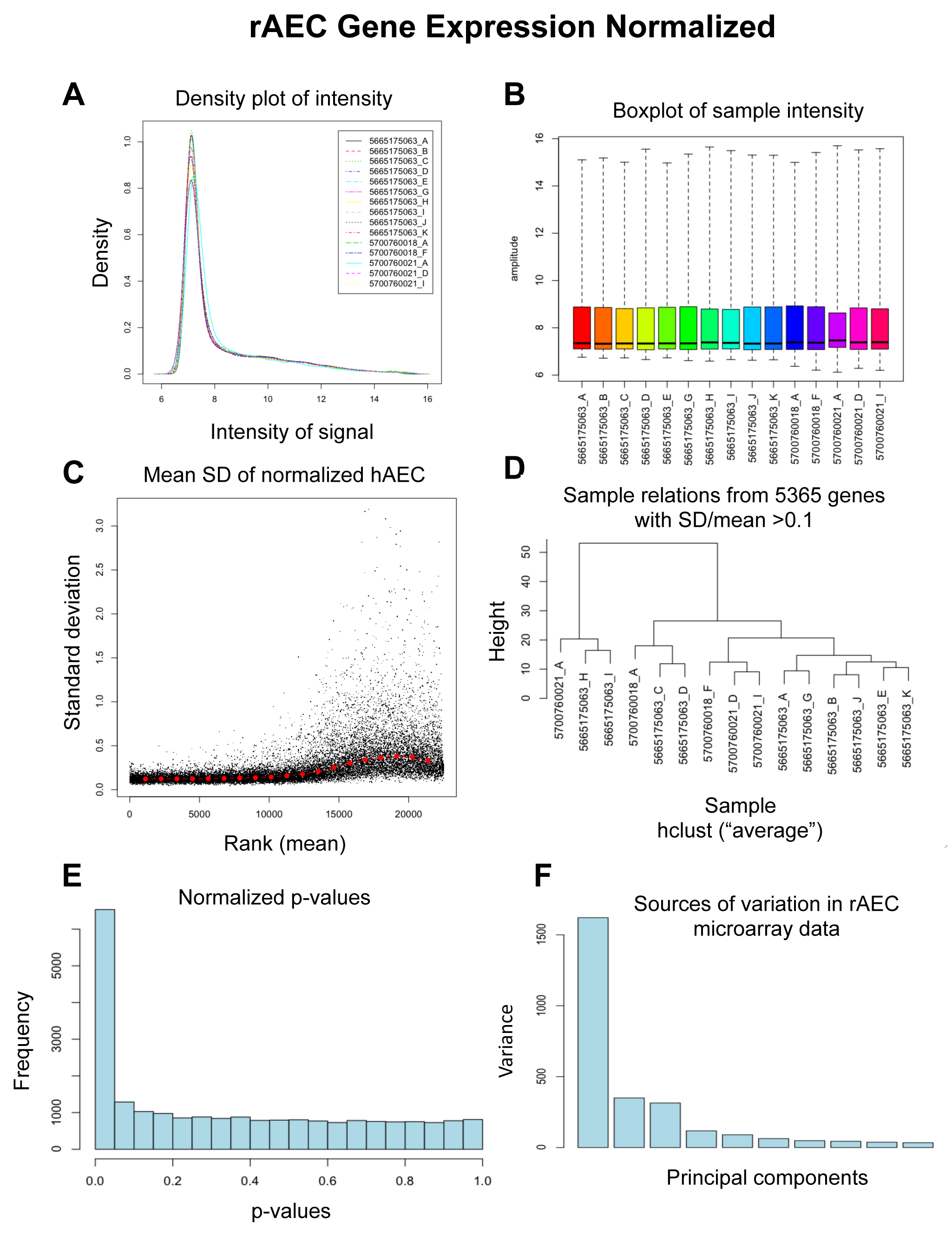

Supplement: Figure S9 — Preprocessed and normalized rat expression profiling. A) Density plot of VSN background-corrected and normalized sample distributions. B) Boxplot of VSN-corrected sample intensity distribution. C) Ranked mean standard deviation of signal for all normalized samples. D) Dendrogram of sample similarity based on top variant genes, those with standard deviation/mean >0.1. E) Distribution of p-values from normalized data (x-axis) and their rate of occurrence (y-axis). F) Principal component analysis of normalized sample data. Each bar represents a source of variation. Bar height indicates amount of variation. (TIF) [file pgen.1003513.s009.tif]

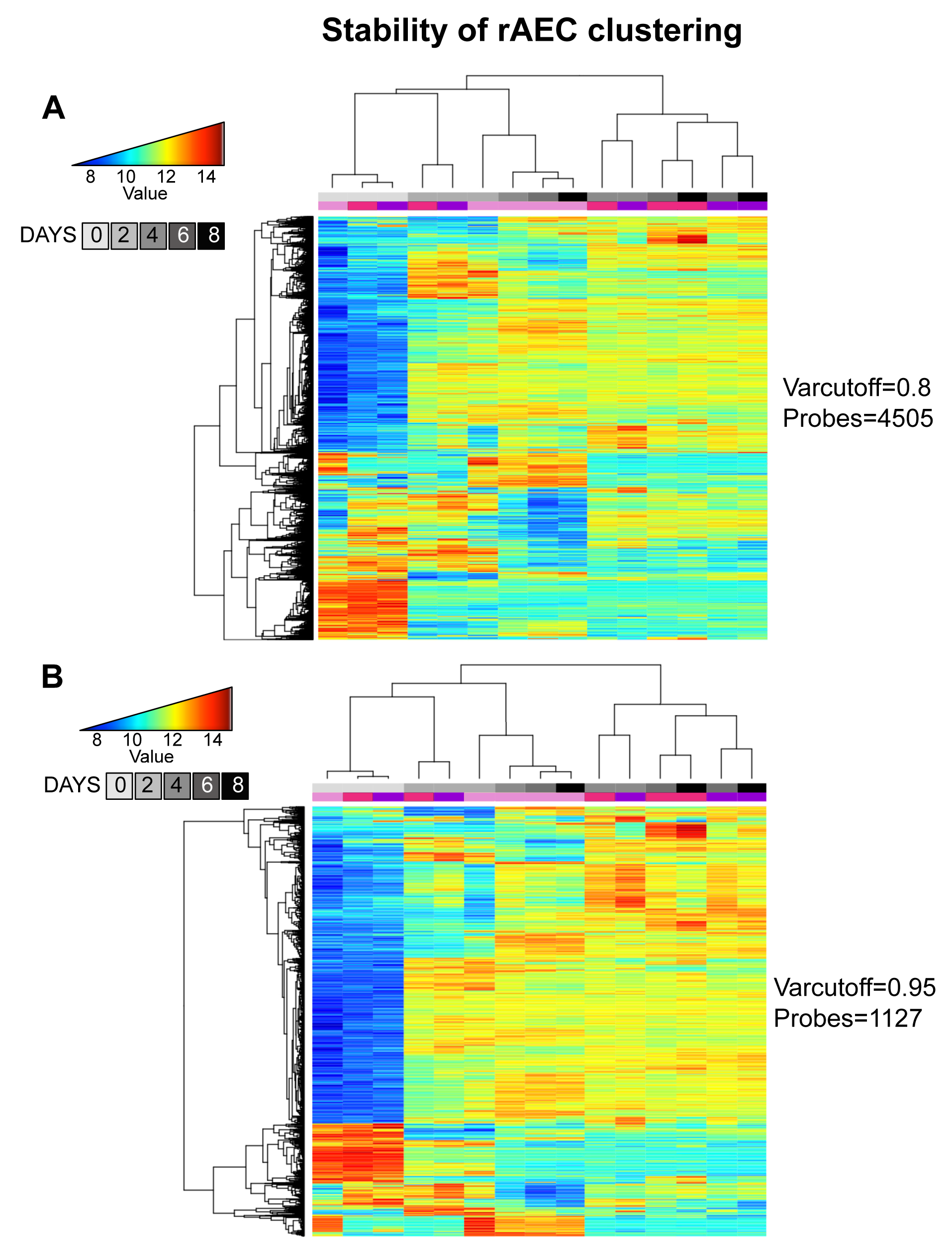

Supplement: Figure S10 — Stability of rat gene expression clusters. A) Heatmap clustering of top 20% of variant genes between samples, clustering using Ward's method. B) Heatmap clustering of top 5% of variant genes between samples, clustering using Ward's method. (TIFF) [file pgen.1003513.s010.tiff]

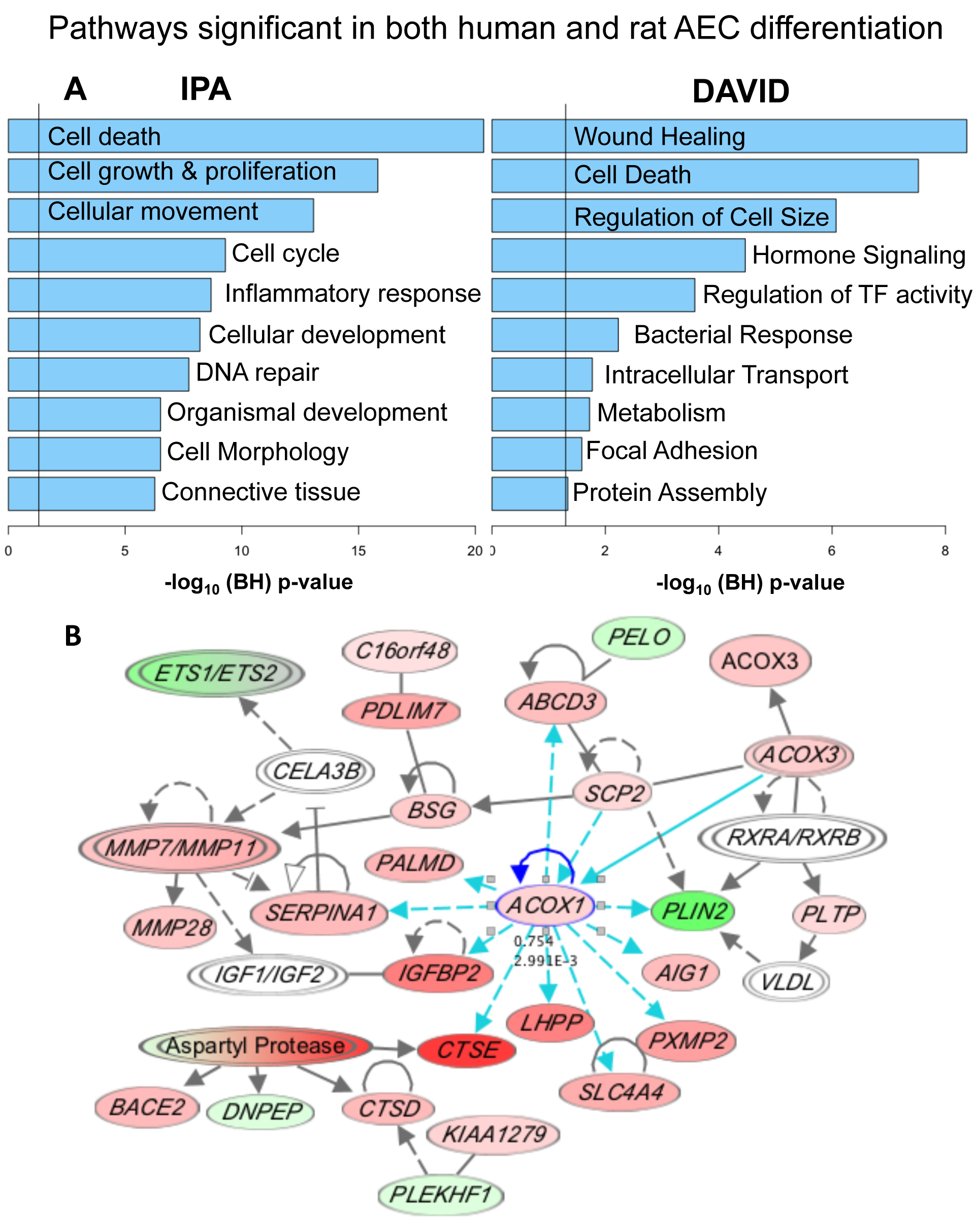

Supplement: Figure S11 — IPA network analysis of overlapping human and rat significant gene expression changes. A) IPA (left) and DAVID (right) of genes with significantly altered expression in both human and rat. Data bars expressed as −log10 of the BH-corrected p-value for enrichment of each pathway as compared to background. B) Top IPA network analysis of overlapping significant genes in human and rat expression. Red = upregulated in AT2 to AT1 cell differentiation, green = downregulated in AT2 to AT1 cell differentiation. Intensity of color is indicative of degree of fold change in expression. (TIF) [file pgen.1003513.s011.tif]

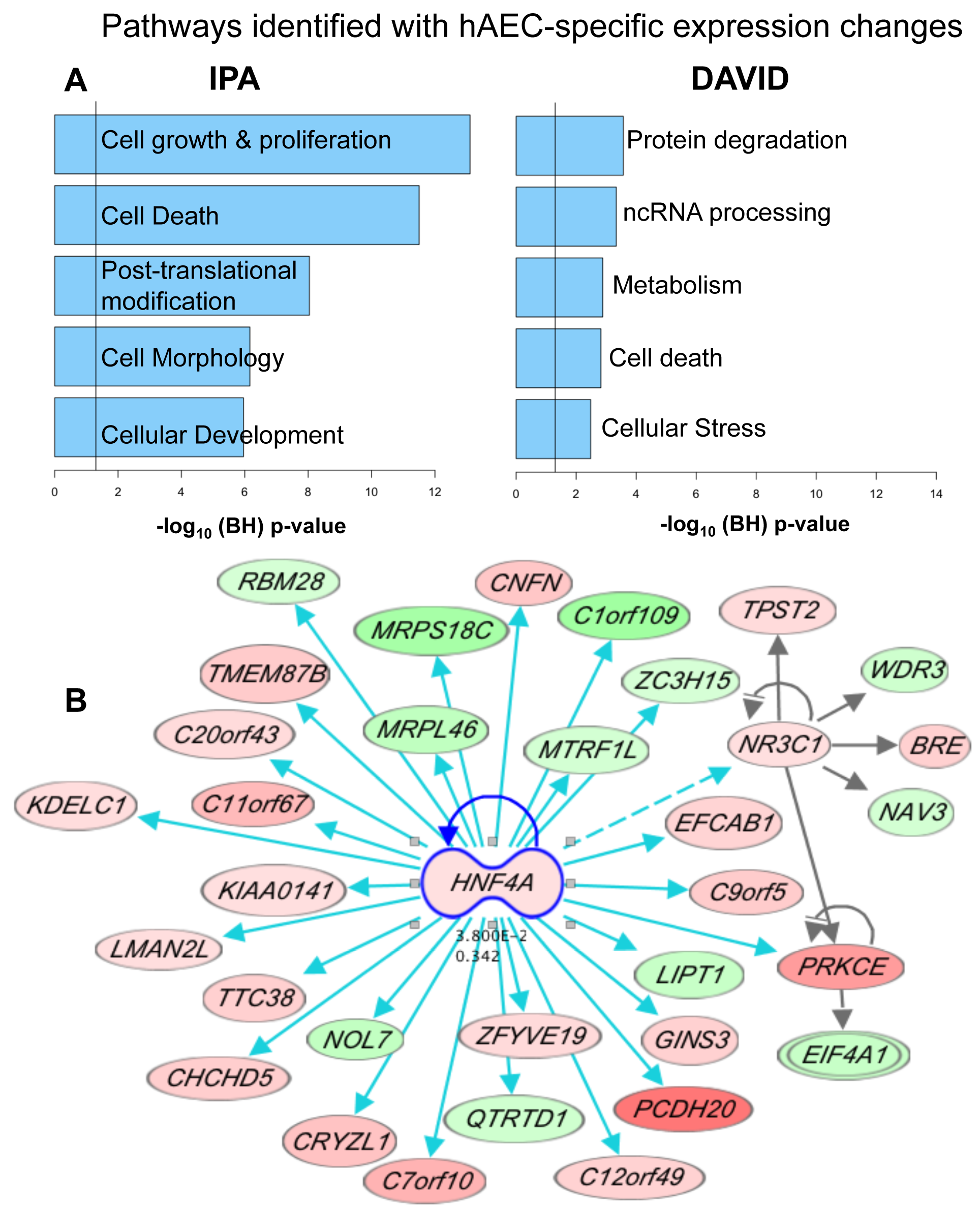

Supplement: Figure S12 — IPA analysis of human-specific gene expression changes. A) IPA (left) and DAVID (right) of human-specific significant gene expression. Data bars expressed as −log10 of the BH-corrected p-value for enrichment of each pathway as compared to background. B) Top IPA network of human-specific changes. Red = upregulated in AT2 to AT1 cell differentiation, green = downregulated in AT2 to AT1 cell differentiation. Intensity of color is indicative of degree of fold change in expression. Blue lines highlight HNF4A connections. (TIF) [file pgen.1003513.s012.tif]

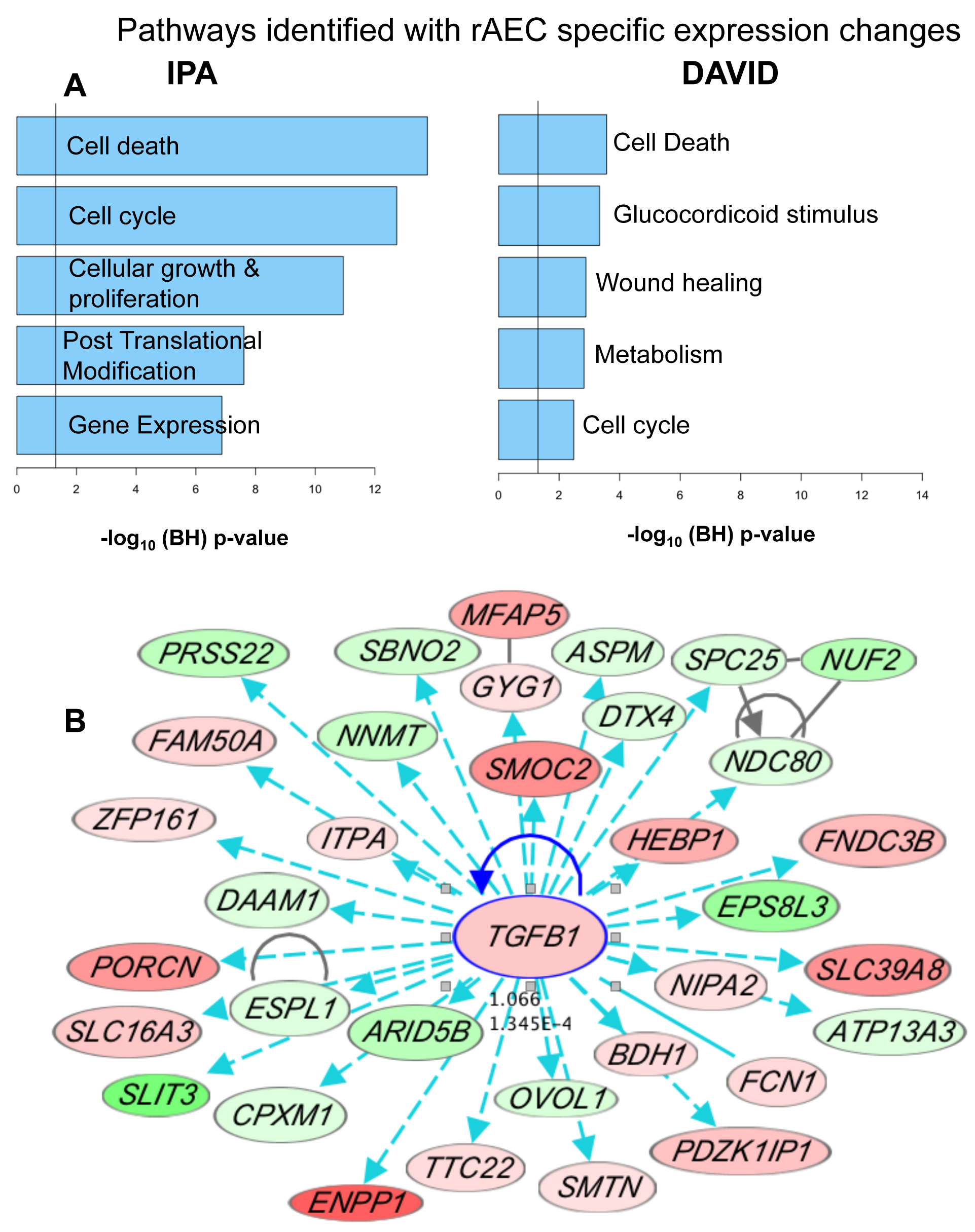

Supplement: Figure S13 — IPA analysis of rat-specific gene expression changes. A) IPA (left) and DAVID (right) of rat-specific significant gene expression. Data bars expressed as −log10 of the BH-corrected p-value for enrichment of each pathway as compared to background. B) Top IPA network of rat-specific changes. Red = upregulated in AT2 to AT1 cell differentiation, green = downregulated in AT2 to AT1 cell differentiation. Intensity of color is indicative of degree of fold change in expression. Blue lines highlight Tgfb1 connections. (TIF) [file pgen.1003513.s013.tif]

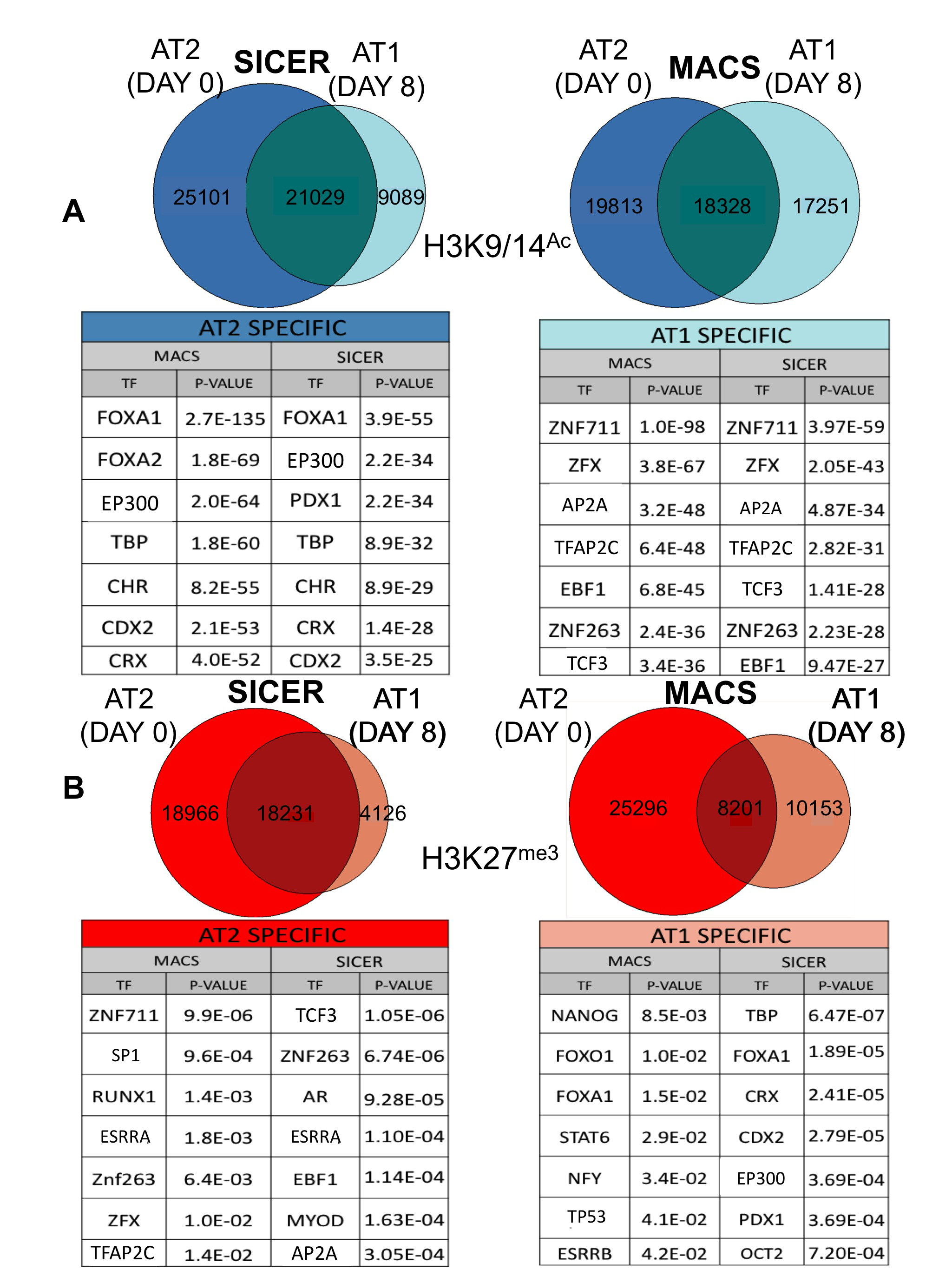

Supplement: Figure S14 — SICER and MACS called peaks and associated predicted TFBS using genomic background. A) SICER and MACS peaks called for H3K19/14Ac chromatin mark. (Middle) H3K9/14Ac peak overlap between chromatin from D0 and D8 (Venn diagram intersection). Left panel: D0-specific chromatin peaks, right panel: D8-specific chromatin peaks B) SICER and MACS peaks called for H3K27me3 chromatin mark. Peak overlap between chromatin from D0 and D8 is at Venn diagram intersection. Left panel: D0-specific chromatin peaks; right panel: D8-specific peaks. For both H3K9/14Ac and H3K27me3, chromatin-enriched DNA motifs are listed and the corresponding transcription factors which bind the recognition sequences in an AT2 (left panels) or AT1 cell-specific fashion (right panels). (TIF) [file pgen.1003513.s014.tif]

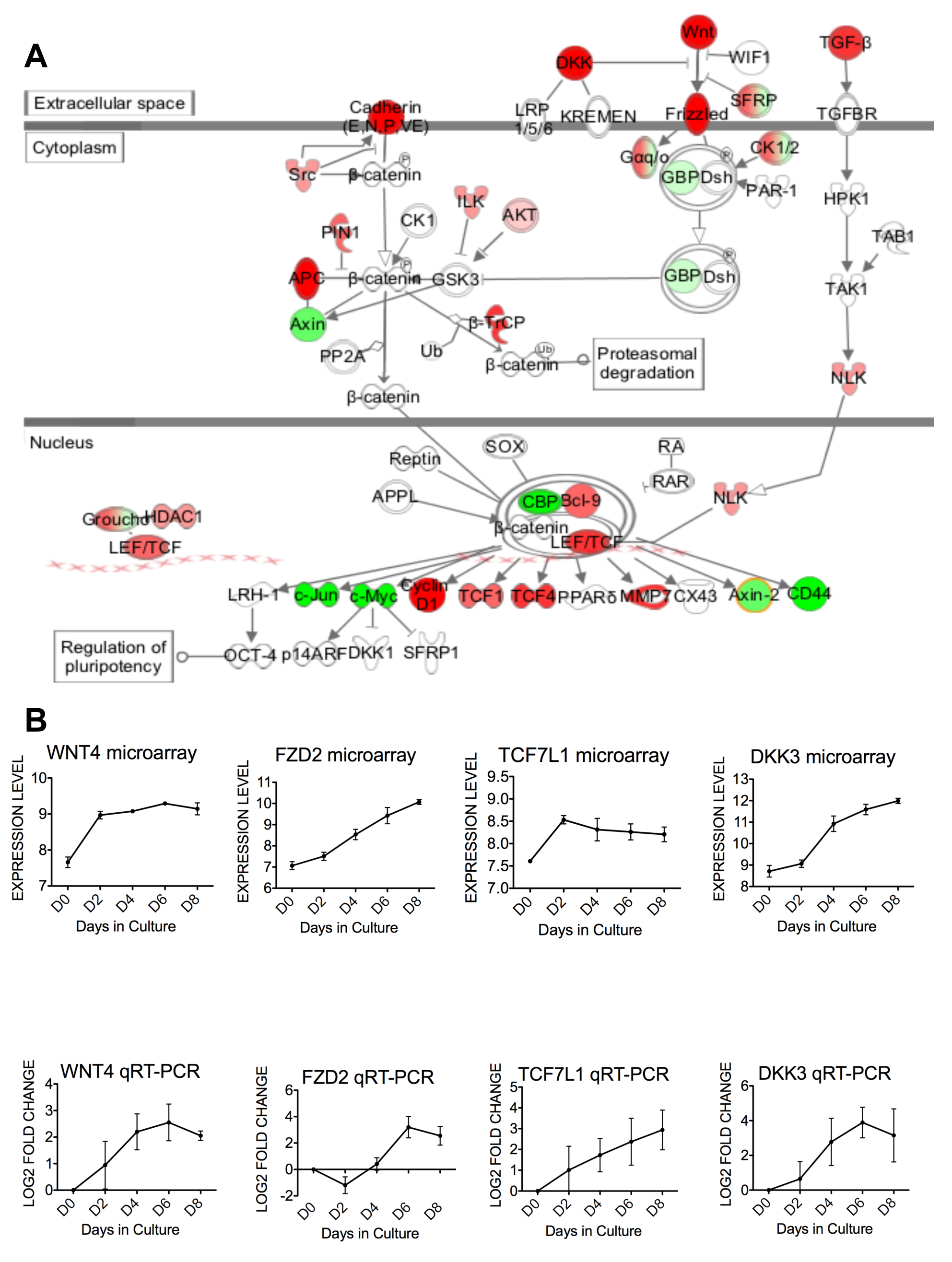

Supplement: Figure S15 — WNT signaling activated in AT2 to AT1 cell differentiation. A) Canonical WNT signaling pathway. Red = upregulated gene expression, green = downregulated gene expression. B) qRT-PCR of select WNT pathway members over course of AT2 to AT1 cell differentiation. 18S rRNA was used for normalization. Error expressed as SEM, n = 3 separate lung donors. (TIF) [file pgen.1003513.s015.tif]

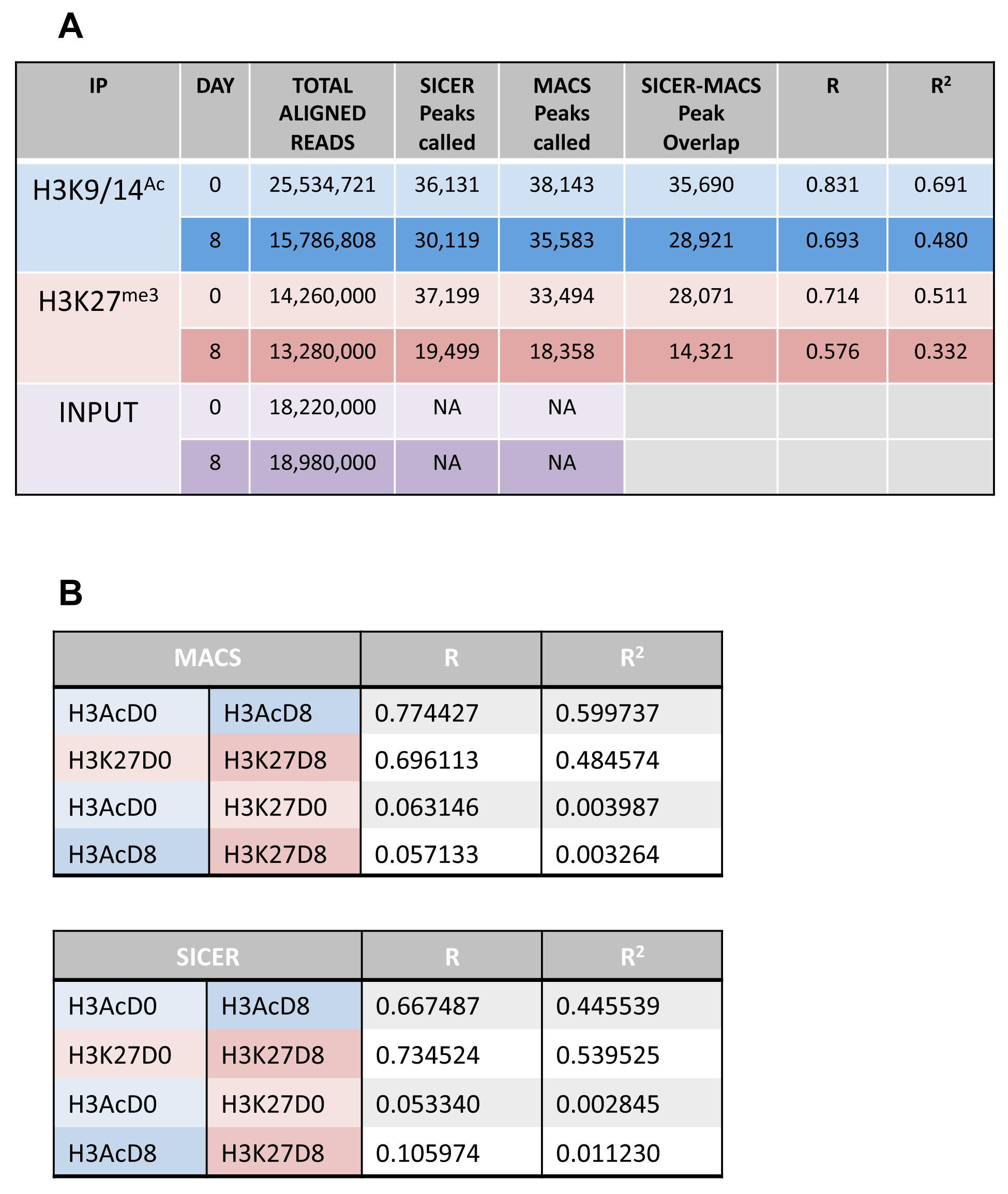

Supplement: Figure S16 — Summary of ChIP-seq data. A) Summary of ChIPs, sequencing data, and peaks called. IP = immunoprecipitation target. R = correlation coefficient. R squared (R2) = degree of correlation. Blue = H3K9/14Ac, red = H3K27me3, purple = input. B) Table demonstrating the degree of correlation between chromatin states between cell types and between differing histone marks. Blue = H3K9/14Ac, red = H3K27me3. (TIF) [file pgen.1003513.s016.tif]

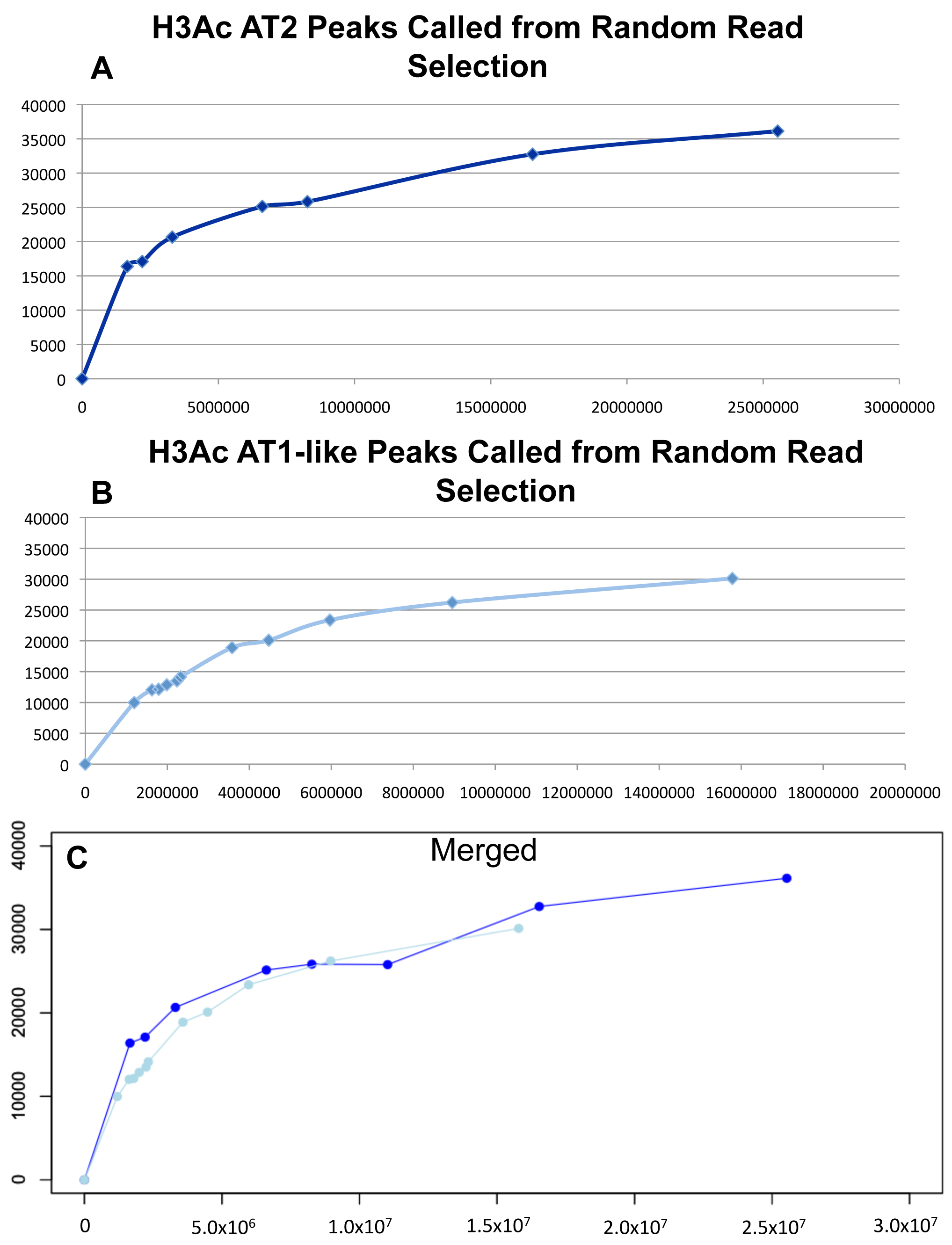

Supplement: Figure S17 — Read count has minimal effect on H3K9/14Ac ChIP-seq peak calling. A) SICER peak calling of H3K9/14Ac in AT2 cells as a function of randomly chosen reads from parent H3Ac AT2 read file. B) SICER peak calling of H3K9/14Ac in AT1 cells as a function of randomly chosen reads from parent H3Ac AT1 read file. C) Merged overlap of peaks called as a function of number of reads included in H3K9/14Ac for both AT2 cells (dark blue) and AT1 cells (light blue). (TIF) [file pgen.1003513.s017.tif]
