# Supplementary material for: Integrated Transcriptomic and Epigenomic Analysis of Primary Human Lung Epithelial Cell Differentiation
Source: PLoS Genet. 2013 Jun 20;9(6):e1003513. doi: 10.1371/journal.pgen.1003513 (PMC3688557; doi:10.1371/journal.pgen.1003513)
Supplement: Table S2 — qRT-PCR primers used in this study. (DOC) [file pgen.1003513.s019.doc]

**Table S2**. **qRT-PCR primers used in this study**

| Target Gene | Forward | Reverse |
| --- | --- | --- |
| 18S | 5’-CAGCCACCCGAGATTGAGCA-3’ | 5’-TAGTAGCGACGGGCGGTGTG-3’ |
| AQP4 | 5’-CATCGCCAAGTCTGTCTTCT-3’ | 5’-AACTCAACCAGGAGACCATGAC-3’ |
| AQP5 | 5’-TTGTGAATGCAGTGCCAAGC-3’ | 5’-GCGGCATTCAATGAACCAGT-3’ |
| BAAT | 5’-ACTTTGGAGAGGTGGTATGTGG-3’ | 5’-AATTCAAGCAGCCCACCCAA-3’ |
| C5 | 5’-TATCCCATCAAGGTGCAGGT-3’ | 5’-ACTCCAGCACCGTCACTCCA-3’ |
| CAV1 | 5’-CAACATCTACAAGCCCAACA-3’ | 5’-TGTGTGTCCCTTCTGGTTCT-3’ |
| CCL20 | 5’-CACTCCCAAAGAACTG-3’ | 5’-GTTGCTTGCTTCTGATTCGC-3’ |
| CDH2 | 5’-ATGTGGACAGGATTGTGGGT-3’ | 5’-TCCCGGCGTTTCATCCATAC-3’ |
| COL1A1 | 5’-AGGGACACAGAGGTTTCAGT-3’ | 5’-CACCAGTAGCACCATCATTT-3’ |
| CST6 | 5’-CTACAACATGGGCAGCAACA-3’ | 5’-GAACCACAAGGACCTCAAAG-3’ |
| CXCL2 | 5’-ACTGAACTGCGCTGCCAGTGCTT-3’ | 5’-TTTCTTAACCATGGGCGATGCGG-3’ |
| DEFB4 | 5’-GATGCCTCTTCCAGGTGTTT-3’ | 5’-GAGACCACAGGTGCCAATTT-3’ |
| DKK3 | 5’-AGGTTGAGGACTGATGGAGGA-3’ | 5’-CTTCGTGTCTGTGTTGGTCTC-3’ |
| FOS | 5’-ACTGATACACTCCAAGCGGA-3’ | 5’-ATCAAGGGAAGCCACAGACA-3’ |
| FZD2 | 5’-TCAGCTACAAGTTTCTGGGC-3’ | 5’-CGCTGCATGTCTACCAAGTA-3’ |
| PDPN | 5’-AACGATGTGGAAGGTGTCAG-3’ | 5’-TCCTGGAGTCACCACATCAT-3’ |
| PGC | 5’-GAAGTGGATGGTGGTGGTCT-3’ | 5’-AAGCGGTACTTCCAAGCAGG-3’ |
| PI3 | 5’-CAAACACCTTCCTGACACCA-3’ | 5’-TTTGACTGGCTCTTGCGCTT-3’ |
| PRSS2 | 5’-ATACAACAGCCGGACTCTGG-3’ | 5’-TTCCAGGGTAGGAGGCTTCA-3’ |
| SCTR | 5’-CATCATGGCCAACTACTCCT-3’ | 5’-GCAATAGCCCACAAAGCAAC-3’ |
| SFTPA2B | 5’-TGAAGGACGTTTGTGTTGGA-3’ | 5’-CAGGAGGACATGGTGTTTCT-3’ |
| SLCO2A1 | 5’-TAGGCCTGCTCATTTCTTCA-3’ | 5’-AATCCACCAGGGAGCCTCTT-3’ |
| SOD2 | 5’-GCTGACGGCTGCATCTGTT-3’ | 5’-CCTGATTTGGACAAGCAGCAA-3’ |
| ST3GAL5 | 5’-TTCTGTTCCAATGCCAAGTG-3’ | 5’-ATCTTGCTTTGAGCTCGGGT-3’ |
| TCF7L1 | 5’-GAAGAAGCCTCTGAATGCCT-3’ | 5’-GAGACAGGTTGTGCCACTTT-3’ |
| TGFB1 | 5’-TCCAAGGTGACAAGCTGGAA-3’ | 5’-GGCTGCAGAACATTGGTGAT-3’ |
| TP63 | 5’-ACAGGAAGGCGGATGAAGAT-3’ | 5’-TGTGTGCTGAGGAAGGTACT-3’ |
| WNT4 | 5’-CCCTCATGAACCTCCACAACAA-3’ | 5’-CTCAGTGGCACCATCAAACT-3’ |
