# Supplementary material for: Integrated Transcriptomic and Epigenomic Analysis of Primary Human Lung Epithelial Cell Differentiation
Source: PLoS Genet. 2013 Jun 20;9(6):e1003513. doi: 10.1371/journal.pgen.1003513 (PMC3688557; doi:10.1371/journal.pgen.1003513)
Supplement: Table S3 — ChIP primers. (DOC) [file pgen.1003513.s020.doc]

**Table S3**. **ChIP Primers**

| Target Promoters | Forward | Reverse |
| --- | --- | --- |
| GAPDH | 5’- GGCTACTAGCGGTTTTACG-3’ | 5’- AAGAAGATGCGGCTGACTGT-3’ |
| MUC4 | 5’- AAACTAGGGACTCCTACTTG-3’ | 5’- GGACAGAATGGGGTGAAT-3’ |
| AQP5 (RXR site) | 5’-GAGAATCAAACCCAGGTCTTCT-3’ | 5’-CTGAGTCAAAGCCAAGGATCT-3’ |
